# Targeting mitochondrial α-ketoglutarate sequestration disables dual oncogenic drivers and metabolic adaptability in pancreatic ductal adenocarcinoma

**DOI:** 10.1038/s41419-026-09016-1

**Published:** 2026-06-25

**Authors:** Yeting Xu, Hongjiang Guo, Shengxi Li, Yucheng Wang, Yinghao Cao, Lin Wang, Qiuxia Chen, Ziyi Qin, Jiaxing Qiu, Yuhan Liu, Yunfan Li, Jiaming Zou, Yingying Wang, Kaihan Zhang, Rui Ju, Lei Guo

**Affiliations:** 1 Department of Pharmacology, Institute of Basic Medical Sciences, Chinese Academy of Medical Sciences & Peking Union Medical College, 100005 Beijing, China; 2 State Key Laboratory of Common Mechanism Research for Major Disease, Institute of Basic Medical Sciences, Chinese Academy of Medical Sciences & Peking Union Medical College, 100005 Beijing, China; 3 Center for Bioinformatics, Institute of Basic Medical Sciences, Chinese Academy of Medical Sciences & School of Basic Medicine, Peking Union Medical College, Beijing, China; 4 State Key Laboratory of Respiratory Health and Multimorbidity, Institute of Basic Medical Sciences, Chinese Academy of Medical Sciences & Peking Union Medical College, 100005 Beijing, China

**Keywords:** Cancer metabolism, Preclinical research

## Abstract

Pancreatic ductal adenocarcinoma (PDAC) exhibits hyperactive mitochondrial metabolism, yet how this rewiring spatially restricts the availability of metabolites for oncogenic signaling and drives systemic metabolic dysregulation in PDAC remains unknown. Here, we identify enhanced mitochondrial α-ketoglutarate (α-KG) sequestration as a key metabolic vulnerability in PDAC. Using multi-omics, preclinical models, and clinical correlation analyses, we identified elevated mitochondrial metabolic gene expression in PDAC. Moreover, higher expression of dihydrolipoamide succinyltransferase (DLST) correlates with poorer PDAC prognosis, suggesting the role of mitochondrial α-KG sequestration in PDAC progression. Targeting mitochondrial respiration with the complex I inhibitor carboxyamidotriazole orotate (CTO) redirected α-KG flux from mitochondrial sequestration, and increased α-KG-dependent m6A demethylation of *MYC* mRNA and HIF-1α hydroxylation. Combining CTO or α-KG dehydrogenase complex inhibitor devimistat with an α-KG analog (dimethyl α-KG) amplified c-Myc/HIF-1α suppression. Consequently, prolonged CTO exposure downregulated multiple metabolic pathways (glycolysis, pentose phosphate pathway, fatty acid synthesis) regulated by c-Myc/HIF-1α, and significantly delayed PDAC progression in vitro and in vivo. Our work first identify a novel mechanism whereby mitochondrial metabolism drives systemic metabolic dysregulation in PDAC through the sequestration of α-KG, and establishes “redirecting α-KG flux from mitochondrial sequestration” as a strategy to disable PDAC’s metabolic adaptability. The orotate salt form of carboxyamidotriazole effectively disrupts mitochondrial α-KG sequestration to suppresses PDAC growth at a dose equivalent to the clinically tested level.

**Figure:**
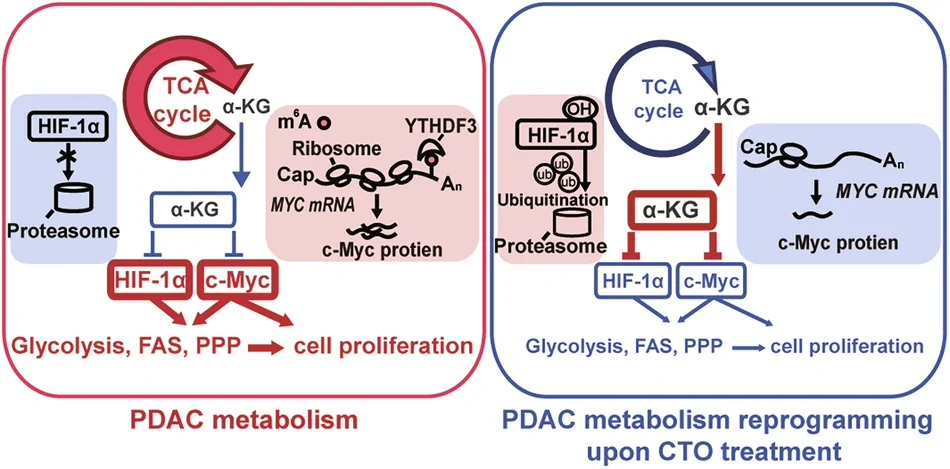

## Introduction

Pancreatic ductal adenocarcinoma (PDAC) remains a lethal malignancy with limited effective treatment options and dismal prognosis. PDAC exhibits multifaceted metabolic aberrations, characterized by enhanced mitochondrial metabolism and upregulation of extramitochondrial metabolic pathways—including glycolysis, glutaminolysis, pentose phosphate pathway, and fatty acid synthesis—driven by oncogenic mutations (e.g., KRAS, TP53) and master transcriptional regulators like c-Myc and HIF-1α [1–8]. Targeting critical metabolic dependencies while disrupting compensatory adaptations may unveil novel, well-tolerated therapeutic strategies.

Notably, PDAC is among the malignancies reported to harbor unique mitochondrial metabolic features, where enhanced oxidative phosphorylation (OXPHOS) fuels tumor progression and therapy resistance [9–11]. Moreover, mitochondrial metabolites serve dual roles as metabolic intermediates and signaling molecules [12–15]. For instance, α-ketoglutarate (α-KG) acts both as a TCA cycle substrate and a co-substrate for epigenetic modifiers and oxygen sensors [16–19]. Beyond its canonical functions in energy metabolism, α-KG serves as an obligatory co-substrate for 2-oxoglutarate-dependent dioxygenases (2-OGDDs) that regulate macromolecular modifications and cellular fate decisions.

OGDD-mediated processes include hydroxylation-dependent degradation of HIF-1α and demethylation of RNA m6A modifications. α-KG-dependent demethylases (e.g., fat mass and obesity-associated protein, FTO) has been reported to demethylate *MYC* mRNA to inhibit its translation [20–22]. In PDAC, both c-Myc and HIF-1α drive tumor progression by orchestrating metabolic pathways to sustain proliferation in nutrient-scarce microenvironments [23–29]. Whether augmenting α-KG-dependent macromolecular modifications can reduce HIF-1α/c-Myc overexpression and reverse systemic metabolic derangements in PDAC remains unexplored. Moreover, no effective pharmacological approach exist to redirect α-KG from mitochondrial catabolism to signaling pools, representing a fundamental gap in targeting PDAC’s metabolic adaptability.

To test whether redirecting α-KG flux could therapeutically reverse systemic metabolic derangements in PDAC, we targeted mitochondrial respiratory chain complex I (CI) - a key regulator of NAD⁺/NADH homeostasis and TCA cycle efficiency. Carboxyamidotriazole orotate (CTO) has been shown to inhibit CI with high safety [30, 31]. CTO may suppress α-KG catabolism in mitochondria, potentially enhancing its extramitochondrial regulatory functions. However, the therapeutic efficacy of clinically achievable CTO doses in PDAC is undefined.

Here we demonstrate for the first time that enhanced mitochondrial α-KG sequestration restricts its function in c-Myc/HIF-1α downregulation and represents a crucial mechanism—apart from oncogenic mutations—driving systemic metabolic hyperactivity in PDAC. We identify “redirecting α-KG flux from mitochondrial sequestration” as an effective strategy to simultaneously suppress c-Myc and HIF-1α to disable PDAC metabolic adaptability. This study is also the first to demonstrate that the orotate salt form of carboxyamidotriazole effectively disrupts mitochondrial α-KG sequestration to suppress PDAC growth in vivo at a dose equivalent to the clinically tested level [32].

## Results

### Mitochondria sequestrate α-KG in PDAC

**Fig. 1:**
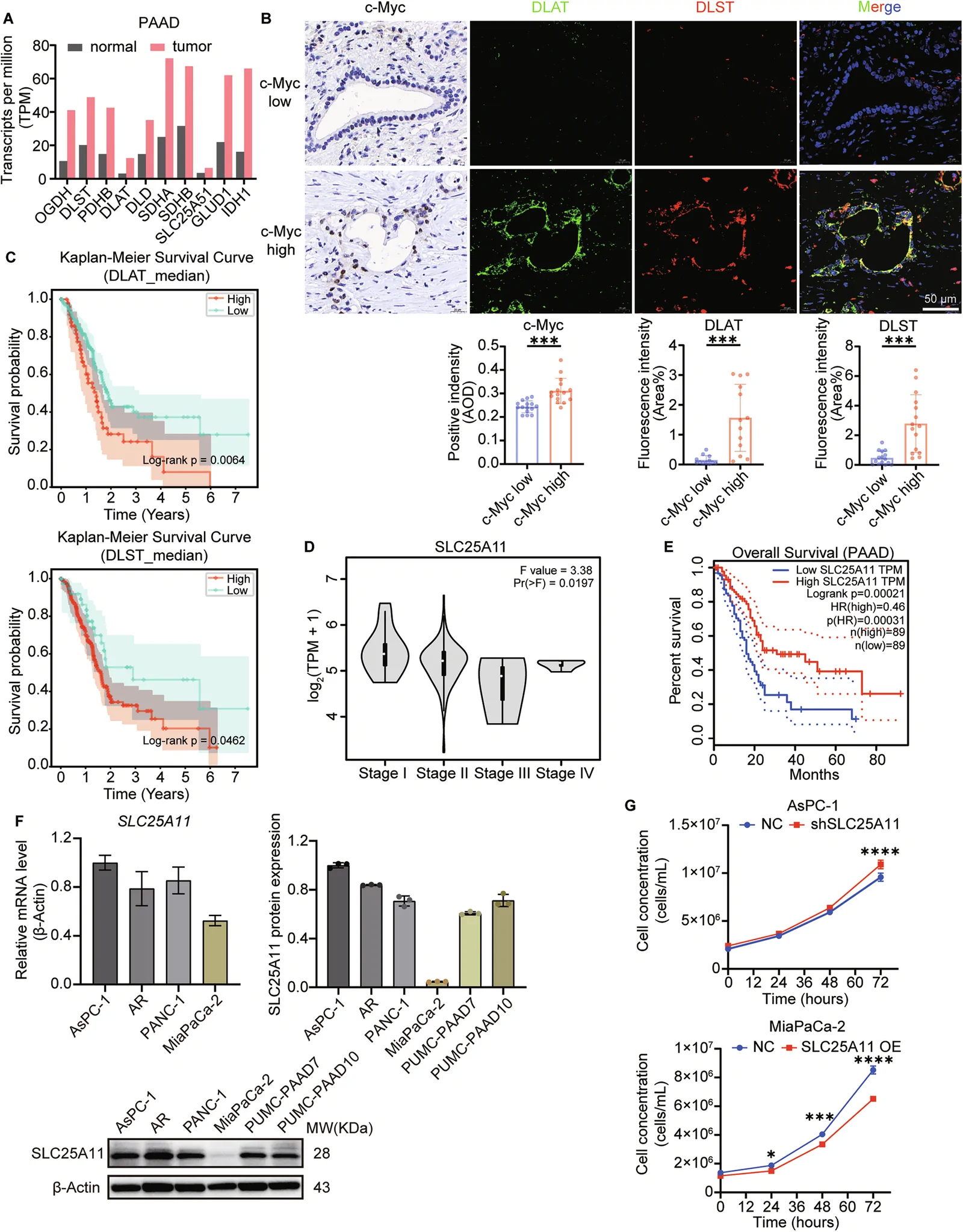
PDAC shows elevated mitochondrial metabolism and mitochondrial α-KG sequestration correlated with progress. **A** TCGA data were analyzed by the GEPIA web server. The expression profiles of mitochondrial metabolism-related genes in PAAD. **B** Immunohistochemical (IHC) or immunofluorescence (IF) staining was performed on the pathological sections of 7 pancreatic cancer patients from Peking Union Medical College Hospital. The patients were serially numbered T1 to T7. IHC staining of c-Myc and IF staining of DLAT and DLST for the same pancreatic ducts of patient T2 (40×) are shown; scale bar = 50 μm. The expression levels of DLAT, DLST, and c-Myc in patients T2, T3, and T7 were quantitatively analyzed. Data represent mean ± SD, *n* = 15 per group, 5 pancreatic ducts per patient. ***, *P* < 0.001 in unpaired *t* test. **C** mRNA expression profiles and corresponding clinical data of DLAT and DLST were obtained from TCGA database. Kaplan–Meier curves show the survival probability in patients with either low or high expression. Statistical evaluation was performed with the log-rank *P* value. TCGA data were analyzed by the GEPIA web server. SLC25A11 mRNA expression profiles in PAAD grades (**D**) and survival curve of PAAD patients (**E**). **F** RT-qPCR analysis of *SLC25A11* mRNA levels and western blot analysis of SLC25A11 protein expression in PDAC cells. Data represent mean ± SD, *n* = 3 per group. **G** Cell growth was evaluated using a Countstar automated cell counter. AsPC-1 cells with SLC25A11 knockdown (shSLC25A11) and MiaPaCa-2 cells with SLC25A11 overexpression (OE) were compared with their respective negative controls (NC) at the indicated time points. Data are presented as mean ± SD, *n* = 3 per group. *, *P* < 0.05, ***, *P* < 0.001; ****, *P* < 0.0001 in two-way ANOVA.

Transcriptomic analysis revealed that mitochondrial metabolic genes are significantly upregulated in pancreatic adenocarcinoma (PAAD, predominantly PDAC) compared to normal pancreatic tissues, ranking among the top elevated pathways across multiple cancer types (top 1–7 among malignancies; Fig. 1A; Fig. S1A). Strikingly, PAAD exhibits the highest tumor-to-normal tissue expression ratio of oxoglutarate dehydrogenase (OGDH), suggesting that pancreatic cancer cells might prioritize α-KG for mitochondrial catabolism, which could result in enhanced mitochondrial α-KG sequestration. Immunohistochemical (IHC) and immunofluorescence (IF) staining of PDAC patient tissues revealed that malignant ductal structures with disordered architecture exhibit concurrent overexpression of c-Myc and mitochondrial enzymes dihydrolipoamide S-acetyltransferase (DLAT)/ dihydrolipoamide succinyltransferase (DLST) (Fig. 1B). High expression of *DLST or DLAT* predicts poor survival in PAAD patients (Fig. 1C).

Moreover, the mitochondrial transporter *SLC25A11*, which transports α-KG out of mitochondria, shows progressive downregulation during PDAC progression, with its expression inversely correlating with tumor grade (Fig. 1D). Intriguingly, *SLC25A11* expression serves as a favorable prognostic marker in PDAC, whereas it has no prognostic value or is negatively correlated with survival in other malignancies (Fig. 1E; Fig. S1B). The altered gene expression of SLC25A11 also affects in vitro PDAC cell growth. We first assessed SLC25A11 expression at both the mRNA and protein levels across PDAC cell lines (Fig. 1F). Based on these results, AsPC-1 cells, which express relatively high levels of SLC25A11, were selected for knockdown, whereas MiaPaCa-2 cells, which express relatively low levels, were selected for overexpression. The efficiency of SLC25A11 knockdown and overexpression was confirmed by western blotting (Fig. S1C). Functionally, SLC25A11 knockdown in AsPC-1 cells increased proliferation, whereas SLC25A11 overexpression in MiaPaCa-2 cells suppressed proliferation (Fig.1G). These findings support a growth-restrictive role for SLC25A11 in PDAC cells and are consistent with the clinical association between low SLC25A11 expression and poor prognosis.

### Mitochondrial α-KG sequestration correlated with c-Myc expression in PDAC

**Fig. 2:**
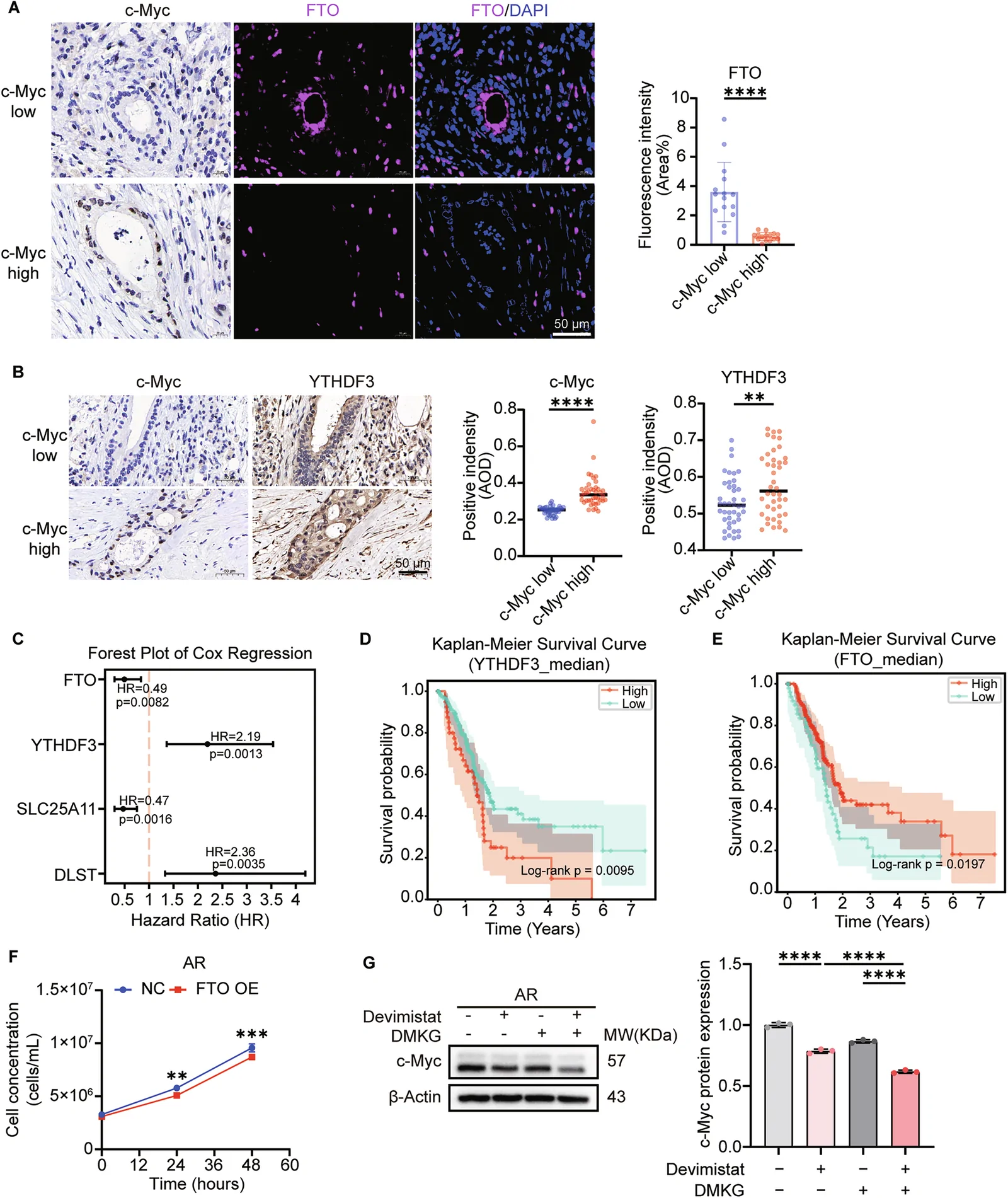
Mitochondrial α-KG sequestration correlated with c-Myc expression and survival period in PDAC. **A** IHC staining of c-Myc and IF staining of FTO for the same pancreatic ducts of patient T2 (×40); scale bar = 50 μm. The expression levels of FTO in patients T2, T3, and T7 were quantitatively analyzed. Data represent ± SD, *n* = 15 in FTO per group, 5 pancreatic ducts per patient. **B** IHC staining of YTHDF3, and c-Myc of patient T2 (20×); scale bar = 50 μm. The expression levels of YTHDF3 and c-Myc in patients T1 to T7 were quantitatively analyzed. Data represent mean ± SD, *n* = 42 per group, 6 pancreatic ducts per patient. **, *P* < 0.01; ****, *P* < 0.0001 in unpaired *t* test. **C** – **E** mRNA expression profiles and corresponding clinical data were obtained from TCGA database. Forest plot of Cox regression shows the prognostic significance of DLST, SLC25A11, YTHDF3, and FTO in PAAD (**C**). Kaplan–Meier curves show the survival probability of YTHDF3 (**D**) and FTO (**E**) in patients with either low or high expression. Statistical evaluation was performed with the log-rank *P* value. **F** Cell growth of gemcitabine-resistant Aspc-1 (AR) cells with FTO overexpression (OE) and negative controls (NC) was assessed at the indicated time points using a Countstar automated cell counter. Data are presented as mean ± SD, *n* = 3 per group. **, *P* < 0.01, ***, *P* < 0.001 in two-way ANOVA. **G**, Western blot analysis of c-Myc in AR cells after treatment with Devimistat (200 μM) for 48 h, and DMKG (5 mM) was added to the culture medium during the last 6 h. Data represent mean ± SD, *n* = 3 per group. ****, *P* < 0.0001 in one-way ANOVA.

α-KG that is not involved in mitochondrial metabolism may participate in reactions mediated by 2-OGDDs, including demethylation of m6A modifications on mRNA. It has been found that decreased m6A on *MYC* mRNA attenuates its translation [17]. We investigated the relationship between the m6A demethylase FTO and c-Myc expression in PDAC. IHC and IF analysis of PDAC tissues revealed inverse correlations of FTO with c-Myc expression (Fig. 2A; S2A).

Conversely, expression of YTHDF3, which recognizes m6A and promotes c-Myc translation, exhibited a positive correlation with c-Myc expression in PDAC samples (Fig. 2B). Complementary bioinformatics analysis through GEPIA revealed that transcriptional levels of *YTHDF3* exhibited strong positive associations with mitochondrial metabolic enzymes and canonical c-Myc target genes (*G6PD*, *SREBF1*, *CDK4*, *MCM6*) in PAAD cohorts (predominantly PDAC) (Table S1). In PDAC, the highest expression ratio of *YTHDF3* (tumor vs. normal) was also found (Fig. S2B).

Integrating these findings, we analyzed the prognostic value of *DLST*, *SLC25A11*, *FTO*, and *YTHDF3* as a combined gene signature. Survival analysis demonstrated that high *DLST* and *YTHDF3* expression correlated significantly with shorter overall survival, whereas high *FTO* and *SLC25A11* expression were associated with significantly longer survival (Fig. 1C; 1E; 2C–E). In FTO-overexpressing PDAC cells, the proliferation rate was also slightly reduced (Fig. 2F). GEPIA-based correlation analyses further demonstrated strong positive associations between mitochondrial metabolic genes (*OGDH*, *DLST*, *PDHB*, *DLAT, DLD*, *SLC25A51*, and *SDHB*) and c-Myc target genes (*G6PD*, *SREBF1*, *CDK4*, *MCM6*; Table S2) [33]. The data indicate that expression levels of genes involved in mitochondrial α-KG sequestration and those regulating/recognizing m6A modifications are significantly correlated with c-Myc expression levels.

However, further research found that elevated expression of SLC25A11 does not downregulate c-MYC. SLC25A11 knockdown in AsPC-1 cells decreased c-Myc protein levels, whereas SLC25A11 overexpression in MiaPaCa-2 cells increased c-Myc protein levels (Fig. S2C). Although increased expression of SLC25A11 mediates the transport of α-KG to the cytoplasm, as an important component in malate—aspartate shuttle (MAS), it may still trap α-KG within the metabolic pathway of MAS, which is coupled with mitochondrial metabolism. Increased SLC25A11 may inhibit cell growth by reducing cytoplasmic malate, which participates in NADPH generation to maintain PDAC cell viability.

In contrast, treatment of PDAC cells with the DLST/DLAT inhibitor Devimistat downregulated c-Myc expression. Co-treatment with the membrane-permeable α-KG precursor dimethyl-α-ketoglutarate (DMKG) potentiated this c-Myc downregulation (Fig. 2G).

### The inhibitor of mitochondrial respiratory chain complex I CTO releases mitochondrially sequestered α-KG

**Fig. 3:**
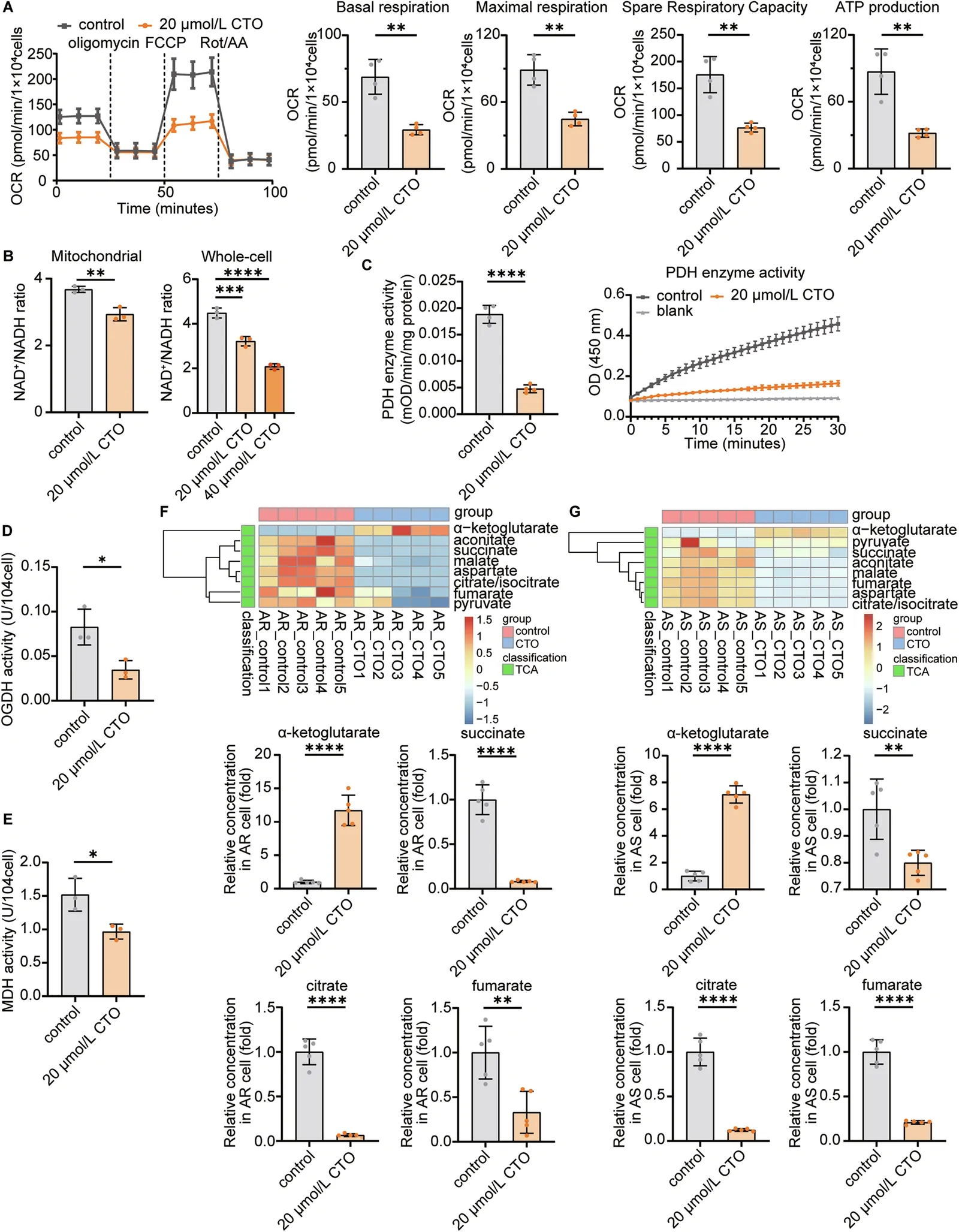
RCCI inhibitor CTO induces mitochondrial metabolic reprogramming. **A** CTO decreased the oxygen consumption rate (OCR) of AR cells. The OCR of AR cells was measured after the sequential addition of oligomycin, FCCP, and Antimycin A plus Rotenone to CTO (20 μM) treatment for 48 h. Data represent mean ± SD, n = 4 per group. **, *P* < 0.01 in unpaired *t* test. **B** The ratio of NAD + /NADH in mitochondria after CTO (20 μM) treatment for 48 h and in whole-cell after treatment with 20 and 40 μM CTO for 6 h. Data represent mean ± SD, *n* = 3. **, *P* < 0.01 in unpaired *t* test. ***, *P* < 0.001; ****, *P* < 0.0001 in one-way ANOVA. The enzyme activities of PDH (**C**), OGDH (**D**), and MDH (**E**) were reduced in AR cells treated with CTO (20 μM) for 48 h. Data represent mean ± SD, *n* = 3 per group. Heatmaps show LC-MS analysis of TCA cycle metabolites in AR (**F**) and AS cells (**G**) treated with CTO (20 μM) for 48 h. Comparison of the abundance of the key metabolites in the TCA cycle between the control and CTO-treated groups. Data represent mean ± SD, *n* = 5 per group. *, *P* < 0.05; **, *P* < 0.01; ****, *P* < 0.0001 in unpaired *t* test.

During our investigation of mitochondrial metabolism, we found an effective approach to break the mitochondrial sequestration of α-KG. The respiratory chain complex I inhibitor CTO significantly induced intracellular α-KG accumulation. In our previous research on PDAC resistance to gemcitabine, we observed enhanced mitochondrial metabolism in gemcitabine-resistant AsPC-1 cells (AR) compared to their gemcitabine-sensitive counterparts (AS) (Fig. S3A–C). We then treated AR cells with CTO and found that CTO significantly reduced OCR (Fig. 3A), decreased NAD + /NADH ratios (Fig. 3B), and suppressed key dehydrogenases’ activity: pyruvate dehydrogenase (PDH), oxoglutarate dehydrogenase (OGDH), and malate dehydrogenase 1 (MDH1; Fig. 3C–E). Metabolomic profiling revealed that CTO treatment depleted TCA cycle intermediates but triggered accumulation of α-KG both in AR and AS cells (Fig. 3F–G).

### CTO inhibits α-KG metabolism within the TCA cycle

**Fig. 4:**
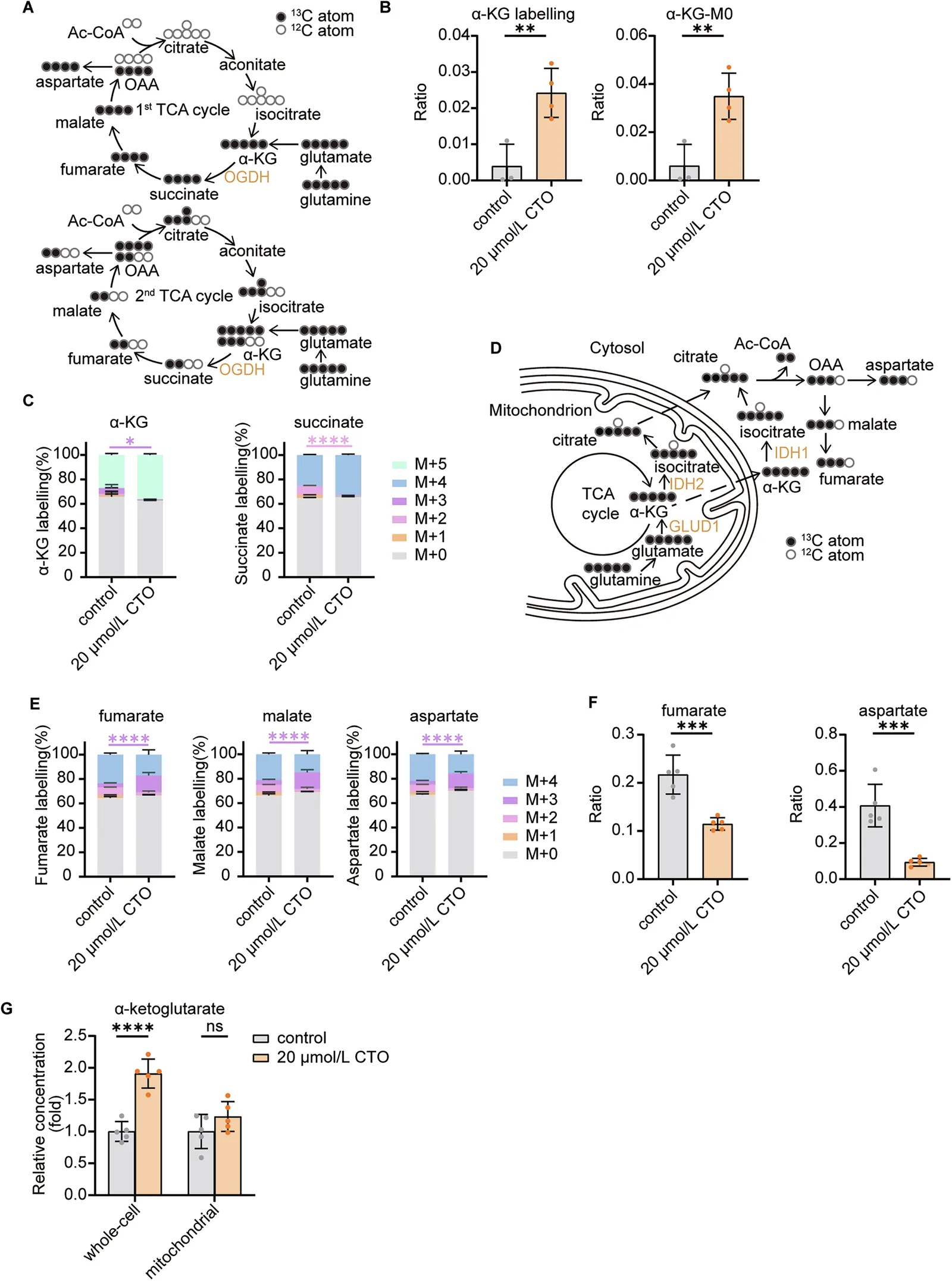
Mitochondrial metabolic inhibition triggers α-KG accumulation. **A** – **F** ^13^C-glutamine metabolic flux assay was used to analyze the flow direction of glutamine in AR cells. Schematic of ^13^C-glutamine metabolic flux in the 1st and 2nd TCA cycles (**A**). Relative abundance of labeled and unlabeled α-KG, *n* = 3 for the control group and *n* = 4 for the CTO(20 μM)-treated group (**B**), and the proportion of succinic acid and α-KG labeling, *n* = 5 per group (**C**). Schematic of ^13^C-glutamine metabolic flux in α-KG reductive carboxylation metabolism (**D**). Comparison of fumaric acid, malic acid, and aspartic acid labeling (**E**), and the relative abundance of fumaric acid and aspartate (**F**) between the control and CTO-treated groups. Data represent mean ± SD, *n* = 5 per group. *, *P* < 0.05; **, *P* < 0.01; ***, *P* < 0.001; ****, *P* < 0.0001 in unpaired *t* test. **G**, LC–MS analysis of whole-cell and mitochondrial α-ketoglutarate levels in AR cells from the control and CTO-treated groups. Data represent mean ± SD, *n* = 5 per group. ****, *P* < 0.0001 in unpaired *t* test; ns not significant.

Given CTO’s inhibition of mitochondrial NAD + -dependent dehydrogenase reactions, we postulated that the accumulated α-KG predominantly originated from glutamine metabolism. Actually, the ^13^C-glutamine metabolic flux assay showed that the levels of both labeled and unlabeled α-KG were increased in CTO-treated cells (Fig. 4A, B). In the 2nd TCA cycle, neither labeled α-KG (m + 3) nor succinate (m + 2) was detected in CTO-treated cells; this finding suggests that the TCA cycle was blocked by CTO treatment (Fig. 4C). Moreover, the proportion of reductive carboxylation of α-KG, indicated by the proportion of m + 3 fumarate, malate, and aspartate was increased in CTO-treated cells (Fig. 4D, E). However, the total levels of fumarate and aspartate were reduced (Fig. 4F), suggesting that reductive metabolism of α-KG were not substantially promoted by CTO treatment, leading to α-KG accumulation. The expression levels of isocitrate dehydrogenase (IDH) 1 and IDH2 were decreased in CTO-treated cells (Fig. S4A). The expression of glutamine metabolism related proteins also suggested that CTO treatment promoted the production of α-KG from glutamine in AR, PUMC-PAAD7, and PUMC-PAAD10 cells (Fig. S4B). The latter two cell lines were established from two Chinese PDAC patients.

Notably, CTO significantly caused an increase in α-KG in whole cells but not within the mitochondria (Fig. 4G), indicating that CTO induced cytosolic α-KG accumulation. Therefore, these data support the proposed model that CTO redirects α-KG availability away from mitochondrial sequestration and increases the α-KG pool accessible to α-KG-dependent enzymes, including FTO.

### CTO downregulates HIF-1α and c-Myc protein levels, potentiated by α-KG supplementation

**Fig. 5:**
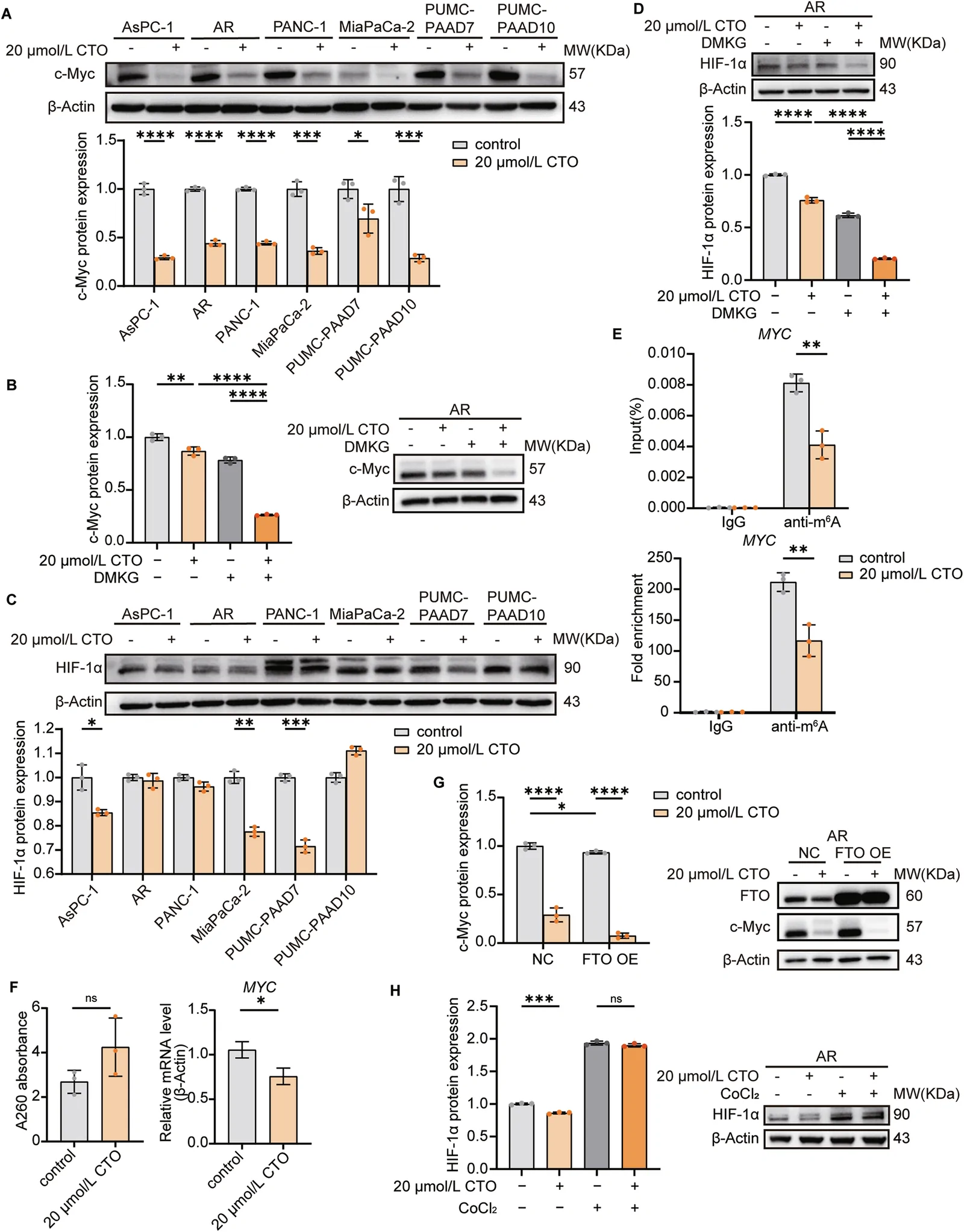
CTO downregulates c-Myc and HIF-1α through α-KG-mediated epigenetic and post-translational modifications. **A** – **D** Western blot analysis of c-Myc in AS, AR, PANC-1, MiaPaCa-2, PUMC-PAAD7, and PUMC-PAAD10 cells in the control and CTO (20 μM)-treated groups (**A**), and in AR cells after treatment with CTO (20 μM) for 48 h, and DMKG (5 mM) was added to the culture medium during the last 6 h of treatment (**B**). The protein levels of HIF-1α were detected under the same conditions as **A** (**C**) and **B** (**D**), respectively. **E** RIP analysis of AR cells with anti-m6A antibodies, followed by RT-qPCR analysis of *MYC* mRNA in the control and CTO-treated groups. **F** The content of ribosomes and *MYC* mRNA level in ribosomes of AR cells in the control and CTO-treated groups. **G** Western blot analysis of FTO and c-Myc in AR cells with FTO OE and NC after 48 h of CTO treatment. **H** Western blot analysis of HIF-1α in AR cells after treatment with CTO for 48 h, and CoCl_2_ (100 μM) was added to the culture medium during the last 6 h. Data represent mean ± SD, *n* = 3 per group. **, *P* < 0.01; ***, *P* < 0.001; ****, *P* < 0.0001 in one-way ANOVA; *, *P* < 0.05; **, *P* < 0.01; ***, *P* < 0.001; ****, *P* < 0.0001 in unpaired *t* test; ns, not significant.

Since c-Myc expression is correlated with mitochondrial metabolic activity and m6A regulator/reader in PDAC, we investigated whether CTO—by redirecting α-KG from mitochondrial catabolism—altered the expression levels of c-Myc and HIF-1α. CTO treatment reduced c-Myc protein levels across PDAC cell lines (Fig. 5A). Combining CTO with dimethyl α-KG (DMKG) synergistically enhanced suppression of c-Myc (Fig. 5B). We further observed that CTO significantly downregulated HIF-1α levels (Fig. 5C). Notably, combining CTO with DMKG also synergistically enhanced the suppression of HIF-1α (Fig. 5D).

Notably, the downregulation of c-Myc and HIF-1α by CTO occurred without altering MYC or HIF-1α transcript levels (Fig. S5A-B), ruling out transcriptional regulation. CTO-mediated c-Myc reduction was independent of phosphorylation-driven ubiquitination. While proteasome inhibition with MG132 elevated total c-Myc and its phosphorylated forms (p-T58 and p-S62) [34–38]. CTO treatment substantially suppressed these protein levels regardless of MG132 co-treatment (Fig. S5C-D).

Given that α-KG promotes m6A demethylases’ activity and was elevated upon CTO treatment, we investigated if CTO regulates α-KG-mediated m6A demethylation in *MYC* mRNA. RIP-qPCR revealed significant reduction of m6A modifications on *MYC* mRNA in CTO-treated cells (Fig. 5E, S5E). Despite a lower decay rate (Fig. S5F), this demethylation correlated with decreased *MYC* mRNA polysome loading (Fig. 5F). Consistently, FTO overexpression itself led to a moderate decrease in c-Myc expression, whereas CTO treatment further suppressed c-Myc more prominently in FTO-overexpressing cells (Fig. 5G). Devimistat treatment also markedly reduced the m6A enrichment on *MYC* mRNA (Fig. S5G).

The regulatory effect of Devimistat on HIF-1α is also consistent with the effect of CTO (Fig. S5H). Pharmacological inhibition of prolyl hydroxylase domain (PHD) enzymes using CoCl_2_ attenuated CTO-induced HIF-1α reduction (Fig. 5H), demonstrating that CTO promotes HIF-1α degradation through PHD activation - an α-KG-dependent hydroxylation process.

### CTO suppresses multiple extramitochondrial metabolic pathways regulated by c-Myc and HIF-1α in PDAC

**Fig. 6:**
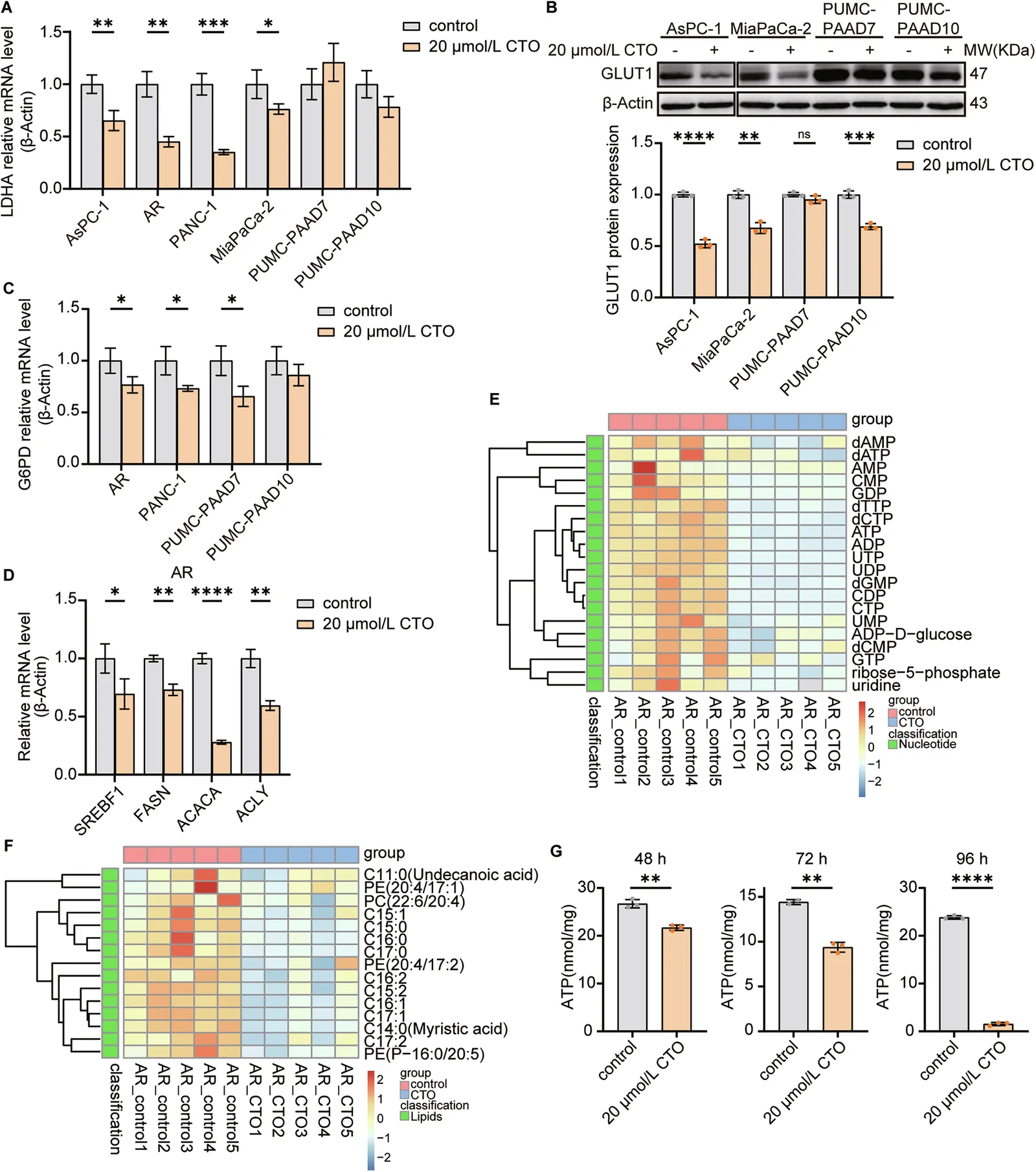
Long-term CTO treatment downregulates extramitochondrial metabolic pathways in PDAC cells. **A** RT-qPCR analysis of LDHA in AS, AR, PANC-1, MiaPaCa-2, PUMC-PAAD7, and PUMC-PAAD10 cells in the control and CTO (20 μM)-treated groups. **B** Western blot analysis of GLUT1 in AS, MiaPaCa-2, PUMC-PAAD7, and PUMC-PAAD10 cells in the control and CTO-treated groups. **C** RT-qPCR analysis of G6PD in AR, PANC-1, PUMC-PAAD7, and PUMC-PAAD10 cells in the control and CTO-treated groups. **D** RT-qPCR analysis of fatty acid synthesis genes in AR cells in the control and CTO-treated groups. Heatmaps show LC-MS analysis of nucleic acid precursors (**E**) and lipids (**F**) in AR cells in the control and CTO-treated groups. **G**, Comparison of ATP levels between the control and CTO-treated groups for 24, 48, and 72 h in AR cells. Data represent mean ± SD, *n* = 3 per group. *, *P* < 0.05; **, *P* < 0.01; ***, *P* < 0.001; ****, *P* < 0.0001 in unpaired *t* test; ns not significant.

Prolonged CTO exposure downregulated glycolytic (*LDHA*, *GLUT1*), pentose phosphate pathway (*G6PD*), and fatty acid synthesis (*SREBF1*, *FASN*, *ACLY*, *ACACA*) genes (Fig. 6A–D; Fig. S6). These genes are direct transcriptional targets of c-Myc and HIF-1α (Table S3). Metabolomic validation showed reduced nucleic acid precursors and lipids (Fig. 6E–F; Fig. S7A-B), confirming extramitochondrial pathways suppression. Failed glycolysis compensation caused progressive ATP depletion (Fig. 6G) and concomitant AMPK pathway activation (increased p-AMPK/p-ACC) (Fig. S7C).

Previous studies have shown that AMPK activation suppresses the expression of c-Myc and HIF-1α [39, 40]. We investigated the temporal patterns of CTO-regulated AMPK activation and the expression of c-Myc and HIF-1α. The results revealed that CTO does not activate AMPK during the early phase of treatment (Fig. S7D), which aligns with the observed compensatory decrease in glycolysis and the time-dependent reduction in ATP levels following prolonged CTO exposure. Furthermore, the downregulation of c-Myc and HIF-1α by CTO occurred no later than AMPK activation (Fig. S7E). These findings suggest that AMPK activation is secondary to the suppression of glycolysis caused by c-Myc and HIF-1α downregulation, rather than being the direct driver of their reduced expression. Notably, AMPK inhibition with compound C failed to rescue CTO-induced growth suppression (Fig. S7F), confirming AMPK activation as a downstream consequence rather than a primary anticancer mechanism.

### CTO suppresses PDAC growth in Vitro and in Vivo

**Fig. 7:**
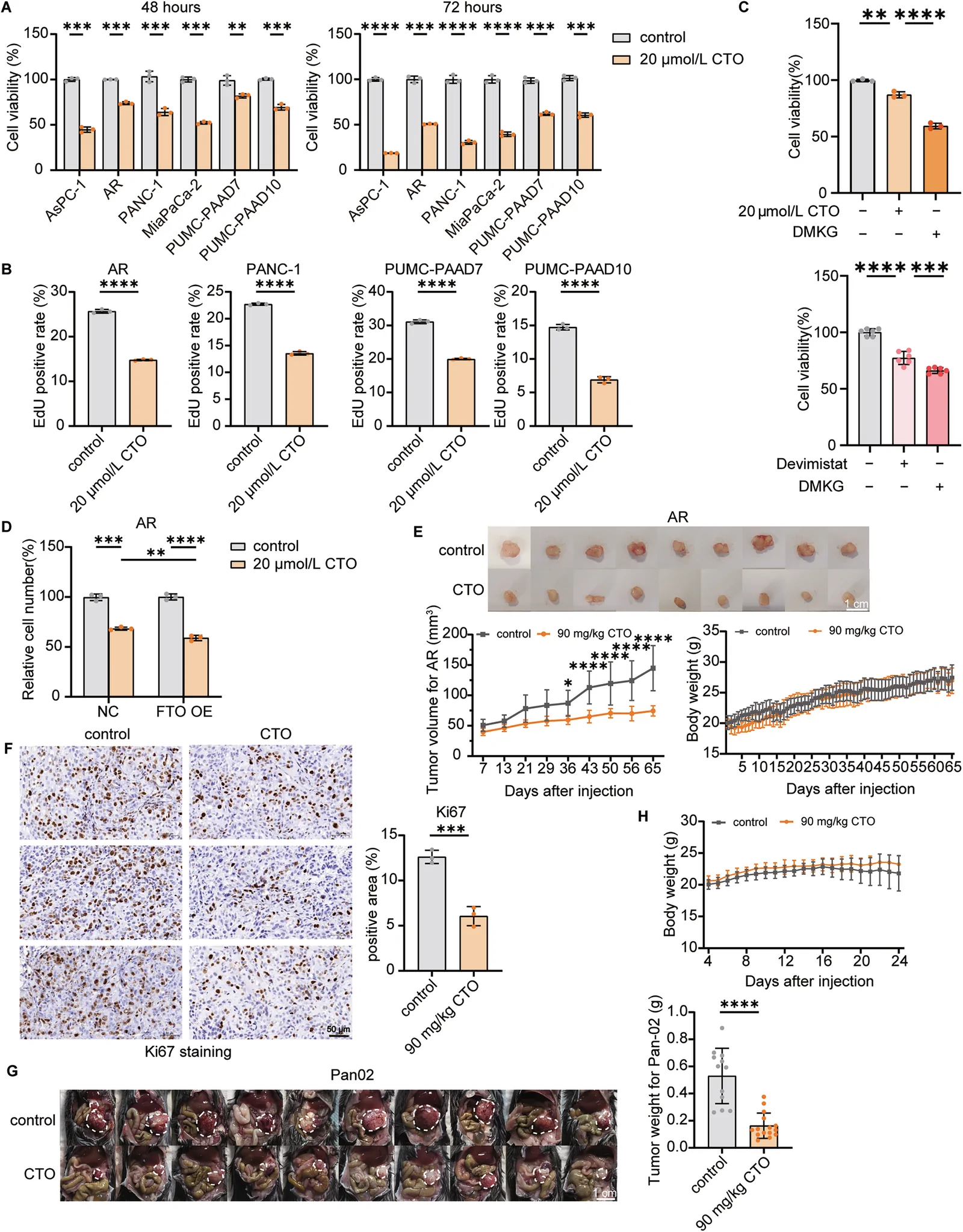
CTO significantly suppresses PDAC growth In vitro and In vivo. **A** Cell viability of AS, AR, PANC-1, MiaPaCa-2, PUMC-PAAD7, and PUMC-PAAD10 cells after treatment with CTO (20 μM) for 48 and 72 h. **B** EdU positivity in AR, PANC-1, PUMC-PAAD7, and PUMC-PAAD10 cells in the control and CTO-treated groups. Data represent mean ± SD, *n* = 3 per group. **, *P* < 0.01; ***, *P* < 0.001; ****, *P* < 0.0001 in unpaired *t* test. **C** Comparison of cell viability among the control, CTO (20 μM) or Devimistat (200 μM)-treated, and CTO or Devimistat plus DMKG (5 mM)-treated groups. Data represent mean ± SD, *n* = 3 per group. **, *P* < 0.01; ***, *P* < 0.001; ****, *P* < 0.0001 in one-way ANOVA. **D** Cell numbers of AR cells with FTO OE or NC were measured using a Countstar automated cell counter after 48 h of CTO treatment and normalized to their respective control groups. Data represent mean ± SD, *n* = 3 per group. **, *P* < 0.01; ***, *P* < 0.001; ****, *P* < 0.0001 in unpaired *t* test. **E** CTO suppresses tumor growth in AR xenograft models in BALB/c nude mice. Data represent mean ± SD, *n* = 8 for the control group and *n* = 11 for the CTO group (90 mg/kg). Comparison of body weight between the control and CTO-treatment groups. Data represent mean ± SD, *n* = 12 per group. *, *P* < 0.05; ****, *P* < 0.0001 in two-way ANOVA. **F** IHC staining of Ki67 in the control and CTO-treated groups (20×) in BALB/c nude mice; scale bar = 50 µm. Data represent mean ± SD, *n* = 3 per group. **G** CTO suppresses tumor growth in orthotopic Pan-02 implantation models in C57BL/ 6 J mice. The areas outlined by the dashed line represents the in situ tumor tissues. Data represent mean ± SD, *n* = 12 for the control group and *n* = 15 for the CTO group (90 mg/kg). **H** Comparison of body weight between the control and CTO-treatment groups in C57BL/ 6 J mice. Data represent mean ± SD, *n* = 13 for the control group and *n* = 14 for the CTO group. ***, *P* < 0.001; ****, *P* < 0.0001 in unpaired t-test.

Proliferation assays demonstrated CTO’s time-dependent efficacy. In detail, live cell counts were reduced following CTO treatment (Fig. 7A). EdU incorporation assays revealed EdU-positive cells was significantly reduced, thus suggesting that DNA synthesis in these cells was inhibited by CTO (Fig. 7B). Combinatorial regimens of CTO or devimistat with DMKG demonstrated potentiated growth suppression in PDAC cells (Fig. 7C). In FTO-overexpressing cells, the inhibitory effect of CTO was further enhanced (Fig. 7D). These enhanced inhibitory effects on proliferation are consistent with a further decrease in c-Myc protein expression.

CTO treatment also significantly delayed the growth of AR cells in vivo, with less extent of Ki67 staining and no apparent influence on body weight of the animals (Fig. 7E–F). Tumor growth was also significantly inhibited in PUMC-PAAD7 and PUMC-PAAD10 xenograft models (Fig. S8A-B). In the orthotopic Pan-02 implantation model, CTO significantly suppressed tumor growth at the pancreatic site (Fig. 7G), and animals administered CTO exhibited higher body weights compared to the control group (Fig. 7H).

### CTO induces metabolic reprogramming and alters extramitochondrial α-KG functions in PDAC

**Fig. 8:**
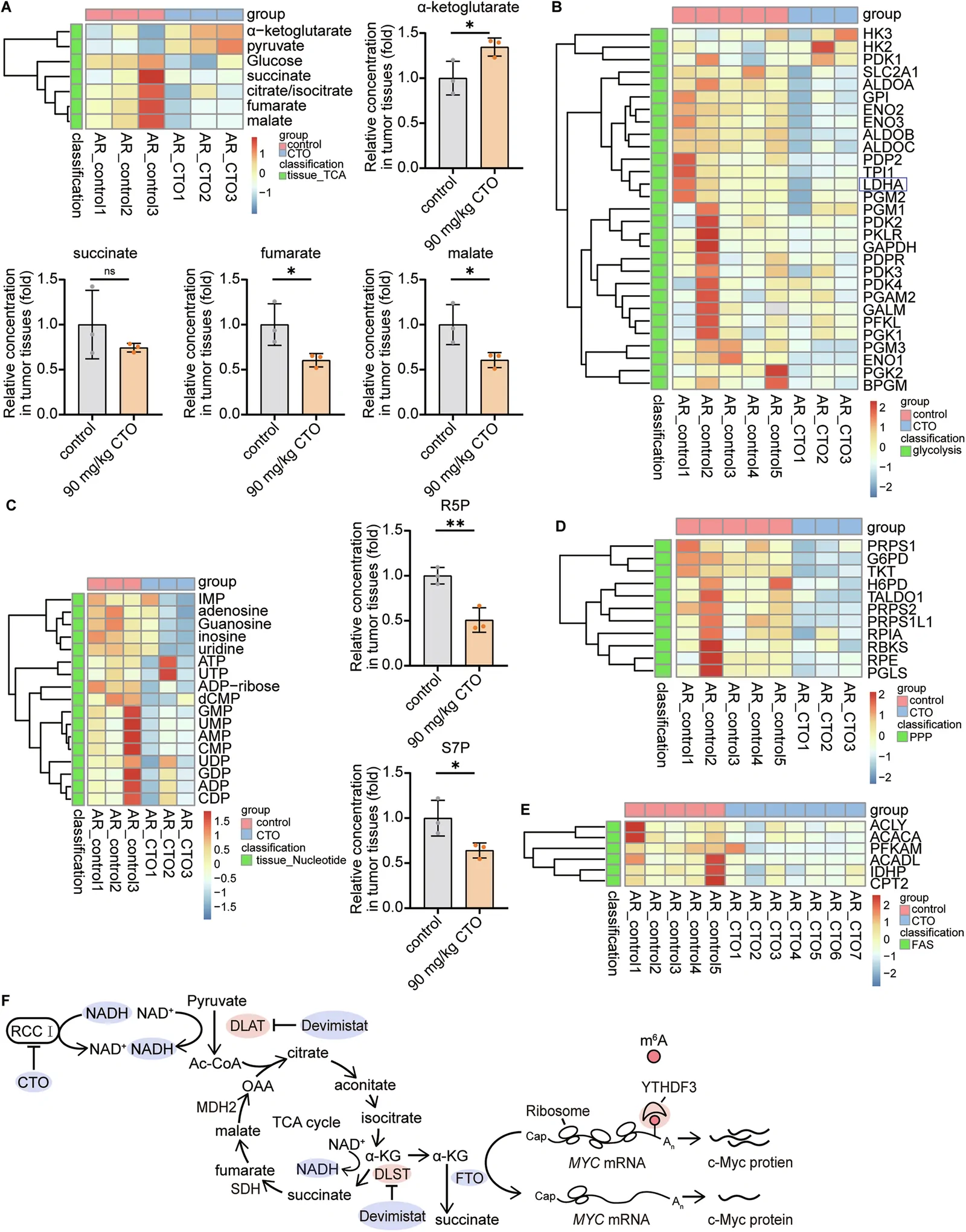
Long-term CTO treatment downregulates metabolic pathways in PDAC tissues by altering extramitochondrial α-KG functions. **A** Heatmap shows LC-MS analysis of TCA cycle metabolites from AR tissues in BALB/c nude mice. Relative abundance of key metabolites in the TCA cycle in the control and CTO (90 mg/kg)-treated groups. Data represent mean ± SD, *n* = 3 per group. **B** Heatmap shows PCR Array analysis of the glycolytic genes from AR tissues in BALB/c nude mice in the control and CTO-treated groups. **C** Heatmap shows LC-MS analysis of nucleic acid precursors from AR tissues in BALB/c nude mice. Relative abundance of R5P and S7P in the control and CTO-treated groups. Data represent mean ± SD, *n* = 3 per group. *, *P* < 0.05; **, *P* < 0.01 in unpaired *t* test; ns, not significant. **D** Heatmap shows PCR Array analysis of the key enzymes of the PPP pathway from AR tissues in BALB/c nude mice in the control and CTO-treated groups. **E** The target protein was analyzed by LC-PRM/MS. The heatmap shows the levels of key enzymes of FAS from AR tissues in BALB/c nude mice in the control and CTO-treated groups. **F** Molecular pattern diagram. The red and blue parts show positive and negative correlation with c-Myc, respectively.

Metabolomic profiling of AR xenograft tissues confirmed that CTO exposure suppressed significantly suppressed TCA cycle intermediates including fumarate and malate, while elevating α-KG levels, consistent with its effect in PDAC cells (Fig. 8A).

long-term administration of CTO reduced c-Myc expression in PUMC-PAAD7 and PUMC-PAAD10 xenografts (Fig. S9A). CTO paradoxically caused pyruvate accumulation without lactate elevation in xenografts (Fig. S9B). This dichotomy correlated with suppressed glycolytic gene expression and reduced LDHA in treated tissues (Fig. 8B). Further metabolic perturbations emerged in pentose phosphate pathway (PPP) regulation. CTO-treated xenografts showed significant reductions in ribose-5-phosphate (R5P), sedoheptulose-7-phosphate (S7P), and other nucleic acid precursors (Fig. 8C), corroborated by downregulation of PPP enzymes in vivo (Fig. 8D). Proteomic analysis revealed broad suppression of fatty acid synthase (FAS)-associated enzymes in CTO-exposed AR tissues (Fig. 8E). These data were consistent with the reduced transcription of PPP and FAS enzymes in CTO-treated PDAC cells.

Consistent with another reported extramitochondrial function of α-KG [14]. 5-hydroxymethylcytosine (5hmC) was significantly increased in CTO treated Pan-02 tumor tissues (Fig. S9C).

These observations propose new regulatory mechanisms in PDAC pathogenesis and therapy: [1] Enhanced mitochondrial metabolism in malignant cells restricts α-KG availability for m6A demethylation of MYC transcripts and hydroxylation of HIF-1α protein to drive the systemic abnormal metabolism, [2] YTHDF3 overexpression facilitates m6A-dependent c-Myc translation, while [3] higher FTO expression or activity promoted by higher α-KG availability (α-KG metabolic inhibition) reduces m6A modification on *MYC* mRNA, consequently attenuating its translational output (Fig. 8F).

## Discussion

α-KG is one of the bifunctional metabolites governing cellular fate [13–16]. It modulates hydroxylation and demethylation reactions that impact oncogenic signaling. Here, we show that PDAC cells exhibit heightened TCA cycle efficiency, compared to normal cells and even other tumor types. Enhanced mitochondrial respiration has been considered to increase oxygen consumption and exacerbates hypoxia [41]. This study shows that elevated mitochondrial metabolism could sequester α-KG toward metabolic flux rather than its role as a cofactor for dioxygenases that suppress oncoproteins like HIF-1α and c-Myc. This creates another regulatory mechanism that complements genetic alterations and hypoxia to sustain elevated HIF-1α/c-Myc levels, thereby amplifying extramitochondrial metabolic pathways and enabling PDAC to maintain a hypermetabolic state that drives proliferation.

Oxidative phosphorylation inhibitors have been shown to alleviate hypoxia in tumor tissues and effectively induce hydroxylation and degradation of HIF-1α even under hypoxic conditions [42–45]. Here, we demonstrate that mitochondrial metabolic inhibition downregulates HIF-1α and c-Myc also by elevating the availability of α-KG. Inhibition of OGDH activity to block α-KG metabolism has been found to promote PDAC differentiation by enhancing TET-mediated elevation of 5hmC levels [15]. This mechanism does not emphasize the impact of PDAC hypoxic environments on TET activity, indicating that the observed increase in TET activity may primarily stem from α-KG accumulation. This further underscores the critical role of α-KG levels in regulating the activity of α-KG-dependent dioxygenases. Part of our experiments were also conducted under hypoxic conditions. The data demonstrate that CTO’s regulatory effects on HIF-1α and multiple metabolic genes remain consistent with those observed under normoxic conditions. Moreover, the metabolic remodeling and downregulation of c-Myc in tumor tissues following in vivo CTO administration recapitulated in vitro findings, and underscored the pivotal role of α-KG in modulating both c-Myc and HIF-1α. As a respiratory chain inhibitor, CTO may also alleviate tumor hypoxia by reducing oxygen consumption, thereby amplifying α-KG-driven epigenetic and post-translational regulation.

Normal cells generate substantially greater total ATP through mitochondrial metabolism compared to cancer cells, reflecting divergent bioenergetic demands: tumor cells allocate ATP primarily for proliferation, stemness maintenance, and metastasis, whereas normal cells require more ATP for specialized functions such as contraction and secretory activity [46]. We propose that this disparity stems from superior nutrient substrate availability in normal cells rather than elevated expression of mitochondrial metabolic enzymes. Supporting this hypothesis, transcriptomic analyses of PDAC patient tissues reveal lower TCA cycle enzyme expression in normal cells compared to their malignant counterparts. This paradox suggests that PDAC tumor cells, despite nutrient limitations, achieve higher TCA throughput efficiency through metabolic rewiring, thereby sequestering α-KG toward energy production at the expense of its regulatory roles. Furthermore, the robust oxidative phosphorylation capacity in normal cells dilutes the metabolic perturbation induced by respiratory chain inhibitors at therapeutic concentrations. This intrinsic buffering mechanism explains the favorable safety profile of such inhibitors, enabling selective disruption of tumor metabolism while preserving normal tissue homeostasis. This may be one of the important reasons why tumor cells are more sensitive to the inhibition of cell proliferation by CTO, which also prevents induction of severe adverse reactions to CTO.

m6A modification can enhance mRNA translation through YTHDF3 binding [47–49]. Previous studies have also shown that m6A modifications on *MYC* mRNA promote c-Myc translation, with the m6A writer METTL3, readers YTHDF1/3, and eraser FTO being involved in this process [20–22]. In this study, we show that YTHDF1/3 increases and FTO decreases in high-grade malignant ductal cells as PDAC progresses. CTO-mediated upregulation of α-KG can activate residual FTO activity, leading to reduced m6A levels in *MYC* mRNA. Future studies are warranted to systematically dissect how FTO genetic manipulation (overexpression or knockdown) modulates CTO’s therapeutic efficacy. Subsequent analyses will evaluate the correlation between the efficacy of CTO and the expression levels of mitochondrial metabolic genes (e.g., DLST, OGDH) and m6A regulatory genes (readers/erasers, e.g., YTHDF1, FTO) in clinical cohorts to uncover predictive biomarkers for therapeutic stratification.

Collectively, this study uncovers mitochondrial α-KG sequestration as a metabolic vulnerability in PDAC, where high expression of TCA cycle enzymes predicts poor prognosis. Releasing α-KG from sequestration by pharmacological redirecting of α-KG flux simultaneously destabilizing HIF-1α and suppressing c-Myc translation to disable tumor metabolic adaptability.

## Materials and methods

### Cell cultures and reagents

Human cell lines include AsPC-1 (AS), PANC-1, and MiaPaCa-2 cell lines (Cell Resource Center, Peking Union Medical College [PCRC], Beijing, China), and gemcitabine-resistant AsPC-1 (AR) cell line (Cellverse) were cultured in DMEM-H (PCRC). Human PUMC-PAAD7 and PUMC-PAAD10 cell lines (PCRC) were maintained in DMEM/F12 (PCRC). Mouse cell line Pan-02 (PCRC) was cultured in DMEM-H (PCRC). The complete cell culture media were supplemented with 10% fetal bovine serum (FBS) (Yeasen, 40131ES), 1% (v/v) penicillin-streptomycin (Yeasen, 60162ES76), and 1% (v/v) glutamine (Solarbio, G0200). The cells were cultured at 37 °C in a humidified environment with 5% CO2 in air. Gemcitabine (3 μg/mL; MedChemExpress, HY-B0003) was incorporated into AR medium to sustain the resistant phenotype, and it was subsequently removed before the experiment. CTO, Devimistat (CPI-613), Compound C (Dorsomorphin), and MG132 were purchased from MedChemExpress (HY-16125, HY-15453, HY-13418A, and HY-13259, respectively). Dimethyl-2-oxoglutarate and CoCl_2_·H_2_O were purchased from Sigma-Aldrich (349631 and 255599, respectively). The cell lines were identified by regular STR profiling during the experiment.

### Human pancreatic cancer tissue specimens

All formalin-fixed paraffin-embedded (FFPE) patient tissue samples were obtained from the Pathology Department of Peking Union Medical College Hospital (PUMCH). Patients diagnosed with pancreatic cancer by PUMCH were eligible for the study, and they were anonymously numbered 1-7. Written informed consent was obtained from all patients. The study was approved by the PUMCH Institutional Review Board (approval number: I-24PJ2172). The clinical characteristics of the 7 patients are shown in Table S4.

### Immunofluorescence (IF) and immunohistochemical (IHC) staining

Multiplexed IF and IHC were performed on FFPE tissues from PDAC patients. For multiplexed IF, the sections were blocked with hydrogen peroxide at room temperature in the dark for 25 min and then incubated with serum or 3% bovine serum albumin for 30 min. The sections were then incubated with the following primary antibodies (Servicebio) overnight at 4 °C according to the specific dilution: anti-DLST (GB114020,1: 5 000), anti-DLAT (GB113649, 1: 2000), and anti-FTO (GB111359,1: 5000). For IHC, the sections were incubated overnight at 4 °C with the following primary antibodies: anti-c-Myc (Abcam, ab32072, RRID: AB_731658, 1: 200) and anti-YTHDF3 (Abcam, ab220161, RRID: AB_2868574, 1: 100). The sections were then incubated with secondary antibodies and subsequently stained with DAB. The nuclei were counter stained with hematoxylin.

The fixed tumor tissues from animals were dehydrated, permeated, paraffin-embedded, and sectioned. As mentioned above for IHC staining, the sections were incubated with the following antibodies: anti-c-Myc (Abcam, ab32072, RRID: AB_731658, 1: 200), anti-Ki67 (ServiceBio, GB121141, RRID: AB_3083641, 1: 600), and anti-5hmC (Proteintech, 39791, 1: 1 000). All sections were scanned and analyzed by CaseViewer software. ImageJ software was used for quantitative analysis.

### Untargeted metabolomics analysis

For whole-cell untargeted metabolomics analysis, 4 × 10^6^ AS and AR cells (*n* = 5) were cultured in 10 cm dishes. After 48 h of CTO treatment, the samples were placed on dry ice and extracted directly by adding 40 μL of precooled 80% (v/v) methanol per 1 × 10^6^ cells, and the cells were scraped off with a cell spatula. The cell solution was centrifuged twice at 15,000 rpm for 30 min at 4 °C. For mitochondrial untargeted metabolomics analysis, mitochondria were isolated by using the Cell Mitochondria Isolation Kit (Beyotime, C3601) according to the manufacturer’s instructions. Metabolites were then extracted from the isolated mitochondria using precooled 80% (v/v) methanol. For mitochondrial metabolomics and the corresponding whole-cell metabolomics samples used for comparison, metabolites were extracted with 20 μL of precooled 80% methanol per 1 × 10⁶ cells. Next, 50 μL of the supernatant was transferred to new injection vials for subsequent high-performance liquid chromatography-tandem mass spectrometry (HPLC-MS/MS) analysis.

For xenograft metabolite analysis, the samples were rapidly frozen in liquid nitrogen. Next, 20 mg of the sample was weighed and ground to powder by a precooled vibrating ball mill (POWTEQ, GT300). Subsequently, the extraction process was performed with 40 μL of precooled 80% (v/v) methanol per milligram of the sample. The extract was centrifuged twice at 15,000 rpm for 30 min at 4 °C. The subsequent process was consistent with the above. Differential metabolite analysis was performed using control and CTO-treated groups based on the following criterion: log2 (fold change) ≥1.

### Gene expression binarization and Cox regression analysis

To streamline the Cox regression modeling, gene expression levels were dichotomized based on the predetermined cutoffs: samples with expression levels above the threshold were assigned a value of 1 (high expression), while those below the threshold were assigned 0 (low expression). Cox proportional hazards regression was then performed to evaluate the prognostic significance of four candidate genes: DLST, SLC25A11, YTHDF3, and FTO.

### Lentiviral transduction and generation of stable cell lines

Stable cell lines were established by lentiviral transduction according to the manufacturers’ instructions. To generate FTO-overexpressing cells, AR cells were transduced with either an empty vector control lentivirus (pLV-CMV-MCS-EF1-Puro) or lentivirus expressing FTO (MailGene, Beijing, China). Similarly, MiaPaCa-2 cells were transduced with either a control lentivirus (EF1α-CMV-copGFP-P2A-Puro) or lentivirus carrying the SLC25A11 overexpression construct (Vigene Biosciences, Shandong, China). For SLC25A11 knockdown, AS cells were transduced with lentivirus carrying either a non-targeting scramble shRNA (pLent-U6-shRNA-CMV-copGFP-P2A-Puro) or an shRNA targeting SLC25A11 (Vigene Biosciences). Stable cells were selected with puromycin, and the overexpression or knockdown efficiencies were validated by western blotting before subsequent experiments.

The shRNA sequence targeting human SLC25A11 was as follows:

5′-TACCCGCCTTGGCATCTATACTTCAAGAGAGTATAGATGCCAAGGCGGGTATTTTTT-3′.

### Western blotting assay

A total of 1 × 10^6^ cells were cultured in 6 cm culture dishes. After cell lysis using 200 μL RIPA lysis buffer (Solarbio, R0020) containing 1% (v/v) PMSF (Solarbio, P0100) and the protein phosphatase inhibitor (Solarbio, P1260), protein concentration was determined with the BCA Protein Quantification Kit (Yeasen, 20201ES86) for normalization. After protein quantification and denaturation with 5× SDS-PAGE Protein Loading Buffer (Yeasen, 20315ES20), the protein expression level of the target gene was quantified. Briefly, proteins were separated on an SDS-PAGE gel prepared with BioSci® New Flash Protein AnyKD PAGE (DAKEWE, 8012011) and transferred onto PVDF membranes (Millipore, IPVH00010). The membranes were then blocked with 5% skimmed milk (Yeasen, 36120ES76) prepared in TBST for 2 h at room temperature and then incubated overnight at 4 °C with the following diluted primary antibodies: anti-β-actin (Cell Signaling Technology, 3700, RRID: AB_2242334), anti-SLC25A11 (Proteintech, 12253-1-AP, RRID:AB_2877840), anti-GLUD1 (Proteintech, 14299-1-AP, RRID: AB_2110515), anti-HIF-1α (Novus, NB100-449, RRID: AB_10001045), anti-c-Myc (Abcam, ab32072, RRID: AB_731658), anti-c-Myc (p-Ser62) (Proteintech, 28915-1-AP, RRID: AB_3085340), anti-c-Myc (p-T58) (Abcam, ab28842, RRID: AB_731667), anti-FTO (Cell Signaling Technology, 45980 T), anti-ACC (Cell Signaling Technology, 3676 T), anti-p-ACC(Ser79) (Cell Signaling Technology, 3676 T), anti-AMPKα (Cell Signaling Technology, 5832, RRID: AB_10624867), and anti-p-AMPKa1 (Cell Signaling Technology, 2537S). This was followed by incubation of the membranes with secondary antibodies at room temperature for 1–2 h, and the immunoreactive bands were observed using an enhanced chemiluminescence kit (Tanon). The relative protein expression levels were quantified by ImageJ software, and the values were normalized with β-actin levels.

### Mitochondrial oxygen consumption and glycolysis rate

Mitochondrial oxygen consumption and glycolysis rate were determined in the control and CTO-treated groups by using a Seahorse Bioscience Extracellular Flux Analyzer (XF24) in accordance with the manufacturer’s protocol. For estimating the mitochondrial oxygen consumption rate, 2.5 × 10^4^ cells were cultured for 48 h, and the oxygen consumption rate (OCR) of cells was measured with the Agilent Seahorse XF Cell Mito Stress Kit (Agilent, 103015-100). Normalization performed with the BCA method (Yeasen, 20201ES86) or by using an automated normalized analysis system (Falcon S300, Alicelligent).

### Mitochondrial extraction and measurement of NAD + /NADH

Cells were cultured in 10 cm dishes and treated with CTO for 48 h. After washing with ice-cold PBS, the cells were harvested to extract mitochondria by using the Cell Mitochondria Isolation Kit (Beyotime, C3601) in accordance with the manufacturer’s instructions and the NAD(H) Quantification Assay Kit (Bioss, AK300) to analyze NAD + /NADH in mitochondria. The levels of NAD + /NADH in whole cells were measured in accordance with the manufacturer’s instructions (BioAssay Systems, E2ND-100).

### Determination of metabolic enzyme activity

The enzyme activities of PDH (Abcam, 109902), succinate dehydrogenase (SDH) (Solarbio, BC0715), and NAD-malate dehydrogenase (NAD-MDH) (Solarbio, BC1045) were measured in accordance with the manufacturers’ instructions. Briefly, whole-cell lysates were mixed with reagents. The absorbance value of the mixture was measured at 450 nm for PDH assay and at 340 nm for SDH and NAD-MDH assays by using a BioTek Synergy Neo2 Hybrid Multimode Reader (Agilent).

### U-^13^C_5_ glutamine stable isotope tracing

A total of 6 × 10^6^ AR cells (*n* = 5) were seeded onto 10 cm culture dishes. The next day, cells were washed three times with PBS and cultured for 6 h in DMEM-H (PCRC) supplemented with 2 mM L-glutamine-^13^C_5_ (MedChemExpress, HY-N0390S1), 1% (v/v) penicillin-streptomycin (Yeasen, 60162ES76), and 10% (v/v) dialyzed FBS (VivaCell, C3820-0050). Then cells were washed three times with ice-cold PBS, and quenched using 40 μL precooled 80% (v/v) methanol per 1 × 10^6^ cells on dry ice. For metabolomics analysis, cell solution was centrifuged twice at 15,000 rpm for 30 min at 4 °C. Additionally, 50 μL of the supernatant was transferred to new injection vials and subjected to HPLC-MS/MS analysis for determining the levels of ^13^C_5_-labeled intracellular metabolites. The ratio represented the fraction of the corresponding section in the M5-labeled ^13^C-glutamine.

### Quantitative reverse transcription-polymerase chain reaction (RT-qPCR)

A total of 1 × 10^6^ cells were cultured onto 6 cm dishes for 48 h with or without CTO treatment. Total RNA was extracted from cells, reversed transcribed, and subjected to RT-qPCR analysis by using *AG RNAex Pro* Reagen, *Evo M-MLV* RT Premix, and SYBR® Green Premix Pro Taq HS qPCR Kit (Accurate Biology, AG21102, AG11706, and AG11701, respectively). The reaction temperature and number of cycles were set in accordance with the manufacturer’s instructions. Target gene expression levels were normalized to the β-actin mRNA level. For the Glucose Metabolism PCR Array plate (Wcgene Biotech, wc-mRNA0162-H), the total reaction volume was adjusted to 10 µL. All primer sequences are listed in Table S5.

### RIP-qPCR and MeRIP-qPCR assays

The Imprint® RNA Immunoprecipitation Kit (Sigma-Aldrich, RIP-12RXN) was used to perform RIP assay. A sufficient number of cells from the control and CTO-treated groups were collected and treated in accordance with the manufacturer’s instructions. Briefly, one-tenth of each lysate was saved as input, and the remaining samples were incubated overnight at 4 °C with magnetic beads pre-bound with anti-m⁶A antibody (Proteintech, 68055-1-Ig, RRID: AB_2918796) or control IgG. The extracted RNA was purified with TRIzol.

For the MeRIP-qPCR assay, total RNA from control and Devimistat-treated cells was fragmented and divided into input and IP fractions at a 1:5 ratio. The IP RNA was immunoprecipitated with magnetic beads pre-bound with anti-m⁶A antibody or control IgG. Enrichment of *MYC* was analyzed by RT-qPCR and normalized to the corresponding input.

### Agarose gel electrophoresis

The samples obtained by RT-qPCR were validated by electrophoresis on a 1.5% agarose gel at 120 V for 30 min. The quantities were determined by ultraviolet spectroscopy.

### Ribosome extraction and profiling

Before cell collection, cycloheximide (CHX) (MedChemExpress, HY-1232) was added to AR culture media at the final concentration of 100 μg/mL for 20 min to stop translation. After washing with ice-cold PBS, 1 × 10^7^ cells from each group were harvested to extract ribosomes by using a Ribosome Extraction Kit (BestBio, BB-36061) in accordance with the manufacturer’s instructions. CHX and RNase inhibitors (LABLEAD, R2000) were added throughout the extraction process. The resulting ribosomes were resuspended in RNase-free water (TransGen Biotech, GI201-01), and the absorbance value was measured at 260 nm by using the NanoDrop One microvolume spectrophotometer (Thermo Fisher). RNA in ribosomes was extracted using TRIzol reagent, reversed transcribed, and subjected to RT-qPCR analysis.

### RNA stability assay

Before cell collection, actinomycin D (MedChemExpress, HY-17559) was added to AR culture media at the final concentration of 5 μg/mL for 0, 1, 2, 4, 6, or 8 h to retain RNA stability. After incubation, total RNA was extracted for reverse transcription and RT-qPCR. The mRNA degradation rate was estimated according to a previous report [50]. After mRNA transcription was blocked by actinomycin D, the Cq value at t = 0 was used to normalize the Cq values at each time point to obtain ∆Cq value (∆Cq = Average Cq of each time point—Average Cq of t = 0). The relative abundance of mRNA at each time point was calculated with the 2^-ΔCq^ method. The mRNA decay rate was determined by a nonlinear regression curve fitting (one phase decay) using GraphPad Prism v. 8.4.0.

### Measurement of ATP levels

The ATP level was measured in accordance with the manufacturer’s instructions (Beyotime, S0026). Briefly, a sufficient number of cells were collected and lysed. Following centrifugation at 12,000 × *g* for 5 min at 4 °C, supernatants were used for assays. Subsequently, ATP standards and working solutions were prepared to detect the ATP levels in the samples. The ATP level was finally normalized by BCA protein quantification.

### Cell growth, cell number, and viability assays

Cell growth was evaluated by direct cell counting. Cells were cultured in 6 cm dishes for different time points. At each time point, cells were harvested by trypsinization, resuspended in culture medium, and counted using a Countstar automated cell counter.

To assess the effect of CTO on FTO-edited cell proliferation, a suitable number of cells were cultured in 6 cm dishes for 48 h with or without CTO. After being harvested by trypsinization and resuspended in culture medium, cells were counted using a Countstar automated cell counter.

Cell viability was determined by the sulforhodamine B assay (Yeasen, 40205ES76). According to standard procedures, cells were seeded onto 96-well plates at the density of 1 × 10^4^ cells per well and cultured for 48 or 72 h. The absorbance value was measured at 515 nm by using a BioTek Synergy Neo2 Hybrid Multimode Reader (Agilent).

### EdU staining

After 48 h of cultivation, cell proliferation was determined using the BeyoClick™ EdU Cell Proliferation Kit with Alexa Fluor 488 (Beyotime, C0071S) in accordance with the manufacturer’s instructions. Briefly, cells were incubated with EdU (10 μM final concentration) for 2 h, fixed with 4% paraformaldehyde at room temperature for 15 min, and permeabilized in Immunol Staining Wash Buffer (Beyotime, P0106). After washing the cells three times in PBS, click additive solution was added, and the cells were incubated for 30 min in the dark. The samples were analyzed using a flow cytometer (BD Accuri™ C6 Plus).

### Xenograft and orthotopic pancreatic cancer models

All animal experiments were approved by Institutional Animal Care and Use Committee (IACUC) of the Institute of Basic Medical Sciences, Chinese Academy of Medical Sciences, and conducted in accordance with the guidelines for the care and use of laboratory animals. All mice were housed under a 12 h light-dark diurnal cycle with controlled temperature (20–25 °C) and relative humidity (40–60%) in the specific pathogen-free-grade animal rearing facility of the Experimental Animal Center, School of Basic Medicine, Peking Union Medical College.

To establish the xenograft mouse model, male mice aged 6–8 weeks were selected for modeling. A total of 5 × 10^6^ AR cells per mouse were resuspended in a 1:1 mixture of Ceturegel® Matrix LDEV-Free gel (Yeasen, 40183ES) and PBS, injected into BALB/c nude (RRID: IMSR_RJ: BALB-C-NUDE) mice. 1 × 10^7^ PUMC-PAAD7 or PUMC-PAAD10 cells per mouse were injected into NOD-SCID (RRID: IMSR_RJ: NOD-SCID) mice. All cells were injected subcutaneously into the flanks. After removal of outlier tumor volume, the mice were randomly divided into two groups. Tumor size was measured twice a week from the sixth day of cell injection, and tumor volume was calculated according to the following formula: V (mm^3^) = 0.5 × long diameter × short diameter^2^. CTO at the dose of 90 mg/kg or the same volume of polyethylene glycol 300 was administered daily by gavage. Before the tumor volume of the control group reached 1000 mm^3^, the mice were euthanized by cervical dislocation method, and tumor tissues were weighed and photographed.

To establish the orthotopic Pan-02 implantation model, C57BL/6 J (RRID: IMSR_JAX: 000664) male mice aged 6–8 weeks were selected for modeling. A total of 1 × 10^6^ Pan-02 cells per mouse were resuspended in 25 μl 1:1 mixture of Ceturegel® Matrix LDEV-Free gel and PBS, injected into the parenchyma of pancreatic tails. The mice were randomly divided into two groups. CTO at the dose of 90 mg/kg or the same volume of polyethylene glycol 300 was administered daily by gavage after 3 days of injection. When the mice in the control group showed significant weight loss, the mice were euthanized, and tumor tissues were weighed and photographed.

Animals with unsuccessful model induction or death before endpoint assessment were not included in the final analysis.

### Targeted proteomics analysis

The xenograft tissue samples were added to SDT lysate, boiled in water for 3 min, ultrasonicated, and centrifuged at 16,000 × *g* for 20 min at 4 °C. The supernatant was collected for the BCA assay. The samples were enzymolyzed using filter aided sample preparation (FASP), desalted with the C_18_ StageTip protocol, and dried under vacuum. A NanoDrop spectrophotometer was used to quantify the redissolved peptide. Then, Shotgun detection was performed. The obtained peptide (2 µg) was analyzed by LC-PRM/MS. Chromatographic separation was performed using the nanoliter flow rate Easy-nLC chromatography system (Thermo Scientific). The following buffer was used: liquid A, 0.1% formic acid aqueous solution; liquid B, mixed solution of 0.1% formic acid, acetonitrile, and water (acetonitrile proportion: 95%). The column was balanced with 95% liquid A. The peptides were isolated and then subjected to targeted PRM-MS using the Q Exactive HF-X mass spectrometer (Thermo Scientific). The resulting RAW MS file was analyzed with the Skyline software v. 4.1 by using the PRM data.

### Data acquisition and preprocessing

The GEPIA (http://gepia.cancer-pku.cn) was used to analyze the data from The Cancer Genome Atlas (TCGA) database for mitochondrial metabolism-related genes and YTHDF3 expressions, correlations between genes, and survival prognosis [33].

The mRNA expression profiles and corresponding clinical data of 176 pancreatic adenocarcinoma samples were obtained from TCGA database. For each gene, the optimal expression cutoff was determined by identifying the threshold that yielded the lowest log-rank *P*-value when stratifying the 176 samples into two groups (high vs. low expression).

### Statistical analysis and visualization

All experimental data were analyzed using GraphPad Prism (v8.4.0) or R software and presented as mean and standard deviation (x ± SD). All survival analyses were conducted using the lifelines Python package (v0.26.4). Kaplan-Meier survival curves and forest plots were generated with matplotlib (v3.3.0) to visualize the association between gene expression and patient outcomes. One-way ANOVA was used for comparison when there were more than two groups, and two-way ANOVA was used to compare the tumor volume and growth curves when there were two groups with multiple experimental days. Unpaired *t* test with two tails was used for comparison between two samples. *P* < 0.05 was considered as statistically significant (*, *P* < 0.05; **, *P* < 0.01; ***, *P* < 0.001; and ****, *P* < 0.0001), and ns denotes not significant.

## Supplementary information

supplementary figures

Table S1

Table S2

Table S3

Table S4

Table S5

uncropped western blot

qPCR RAW DATA

## Data Availability

The original data of the Western blot and qPCR have been included in the relevant attachments. Further information and details are available from the corresponding author on reasonable request.
