# Supplementary material for: Targeting mitochondrial α-ketoglutarate sequestration disables dual oncogenic drivers and metabolic adaptability in pancreatic ductal adenocarcinoma
Source: Cell Death Dis. 2026 Jun 25;17(1):788. doi: 10.1038/s41419-026-09016-1 (PMC13558746; doi:10.1038/s41419-026-09016-1)

Figure 1F

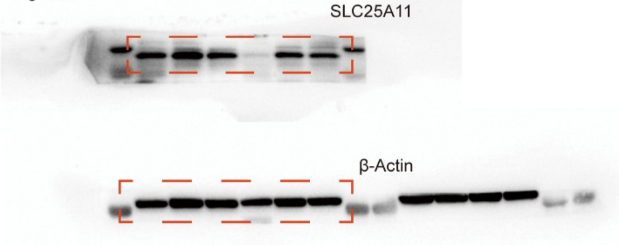

Figure 2G

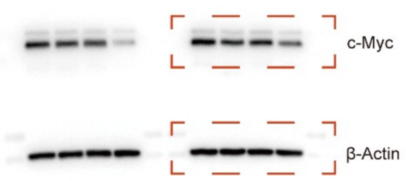

Figure 5A

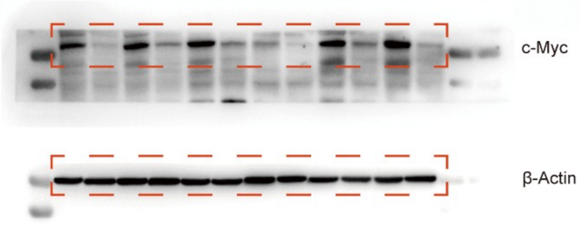

Figure 5B

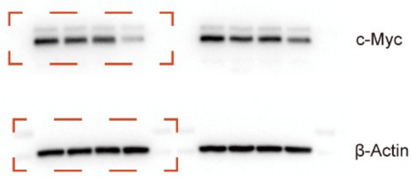

Figure 5C

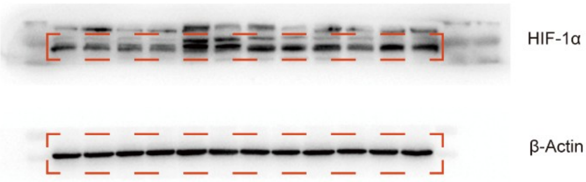

Figure 5D

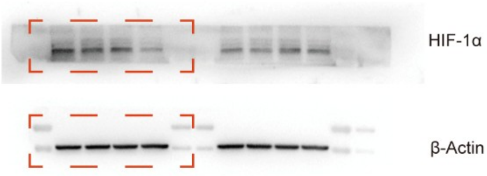

Figure 5G

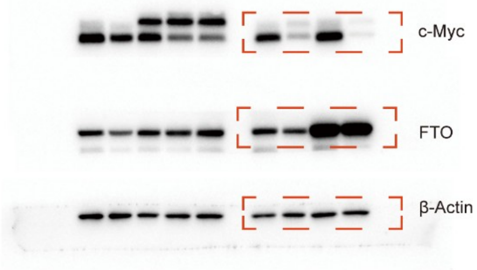

Figure 5H

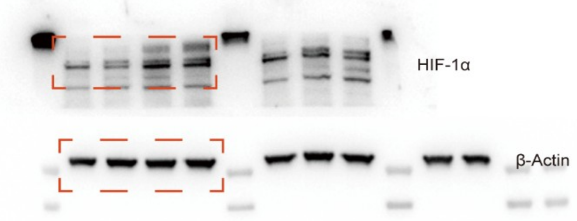

Figure S1C, S2C

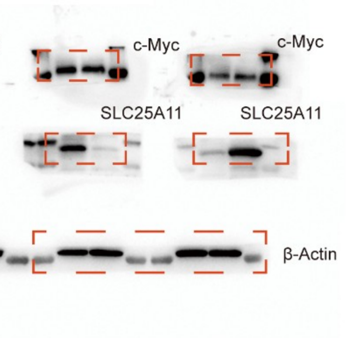

Figure 6B

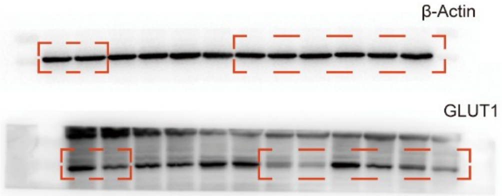

Figure S4B

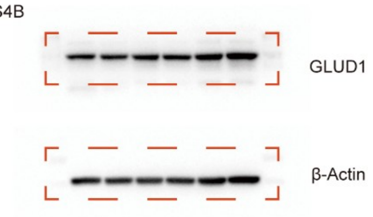

Figure S5C

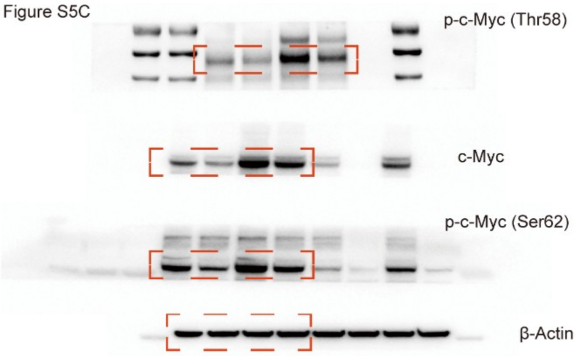

Figure S5D

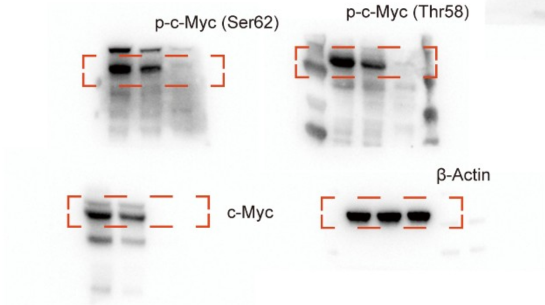

Figure S5H

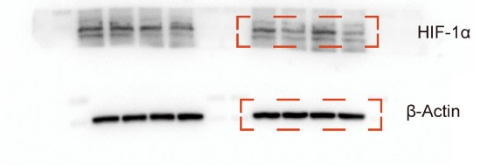

Figure S7C

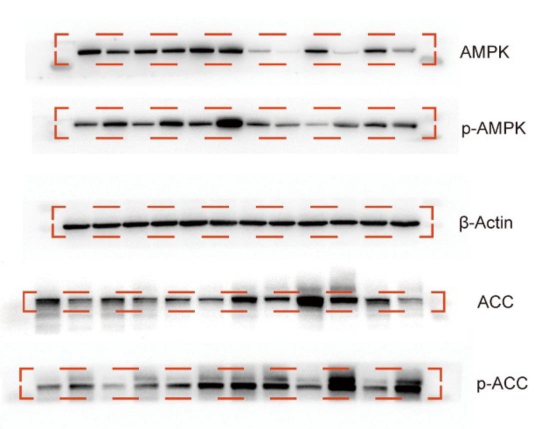

Figure S7D

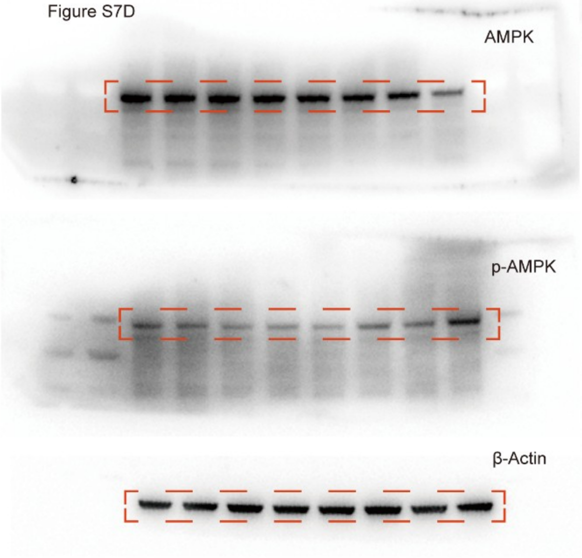

Figure S7E

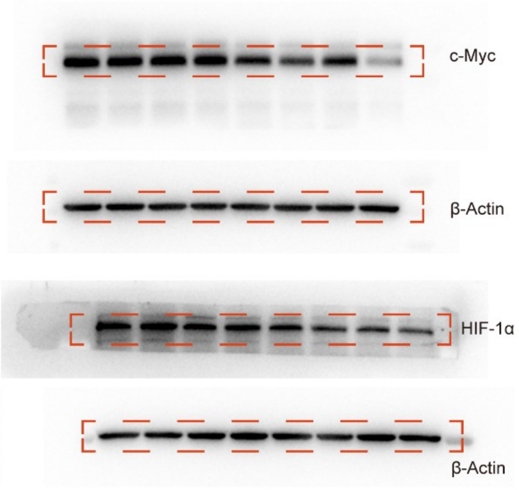

Supplement: Supplementary file 1 [file 41419_2026_9016_MOESM7_ESM.docx]
