# Supplementary material for: Targeting mitochondrial α-ketoglutarate sequestration disables dual oncogenic drivers and metabolic adaptability in pancreatic ductal adenocarcinoma
Source: Cell Death Dis. 2026 Jun 25;17(1):788. doi: 10.1038/s41419-026-09016-1 (PMC13558746; doi:10.1038/s41419-026-09016-1)
Supplement: Supplementary file 1 [file 41419_2026_9016_MOESM1_ESM.docx]

**Figure S1. Mitochondrial metabolic gene expression profiles and mitochondrial α-KG sequestration in malignancies, and validation of SLC25A11 Genetic Manipulation in PDAC Cells. A** and **B,** TCGA data were analyzed by the GEPIA web server. Mitochondrial metabolic gene expression profiles (tumor/normal) (**A**) and SLC25A11 survival curves (**B**) in malignancies. **C,** Western blot analysis of SLC25A11 in AsPC-1 cells with SLC25A11 knockdown (shSLC25A11) and MiaPaCa-2 cells with SLC25A11 overexpression (OE). Data represent mean ± SD, n = 3 per group.

**Figure S2. Clinical relevance of FTO and c-Myc expression in PDAC, YTHDF3 expression across malignancies, and SLC25A11 functions in PDAC cells. A,** Representative images of c-Myc IHC staining and FTO IF staining show different views of the ducts (40×); scale bar = 100 μm; and the same field view of ducts (10×); scale bar = 200 μm. **B,** TCGA data were analyzed by the GEPIA web server. The expression profiles of YTHDF3 (tumor/normal) in malignancies. **C,** Western blot analysis of c-Myc in AsPC-1 cells with SLC25A11 knockdown (shSLC25A11) and MiaPaCa-2 cells with SLC25A11 overexpression (OE), compared with their respective negative controls (NC). Data represent mean ± SD, n = 3 per group. *, *P* < 0.05; ***, *P* < 0.001 in unpaired t-test.

**Figure S3. AR Cells Display Enhanced Mitochondrial Respiration and TCA Cycle Activity Compared with AS Cells. A,** Cell viability in different gemcitabine concentrations and IC_50_ values of AR and AS cells treated for 72 h. Data represent mean ± SD, n = 3 per group. **B,** The OCR of AR and AS cells was measured after the sequential addition of oligomycin, FCCP, and Antimycin A plus Rotenone. Data represent mean ± SD, n = 4 per group. **C,** LC-MS analysis of TCA cycle metabolites (heatmap) and mitochondrial α-ketoglutarate levels in AR and AS cells. Data represent mean ± SD, n = 4 per group. *, *P* < 0.05 in unpaired t-test.

**Figure S4. CTO regulates α-KG production and reductive carboxylation related genes. A,** RT-qPCR analysis of mRNA levels of *IDH1* and *IDH2* in AR cells in the control and CTO (20 μM)-treated groups. **B,** RT-qPCR analysis of mRNA levels of *GLS* and *SLC7A5*, and western blot analysis of GLUD1 protein levels in AR, PUMC-PAAD7, and PUMC-PAAD10 cells in the control and CTO-treated groups. Data represent mean ± SD, n = 3 per group. *, *P* < 0.05; **, *P* < 0.01; ***, *P* < 0.001 in unpaired t-test.

**Figure S5. CTO doesn’t affect the mRNA levels of *MYC*/*HIF-1α* and the stability of c-Myc protein, while altering m^6^A level and stability of *MYC* mRNA. A** and **B,** RT-qPCR analysis of *MYC* in AS, AR, PANC-1, MiaPaCa-2, PUMC-PAAD7, and PUMC-PAAD10 cells (**A**) and HIF-1α in AR and AS cells (**B**) in the control and CTO (20 μM)-treated groups. Data represent mean ± SD, n = 3 per group. **, *P* < 0.01 in unpaired t-test; ns, not significant. **C** and **D**, Western blot analysis of c-Myc and p-c-Myc in AR cells after treatment with CTO for 48 h, without (**C**) or with DMKG (5 mM) (**D**) in last 6 h, and MG132 (10 μM) was added to the culture medium during the last 6 h. **E,** Agarose gel electrophoresis was performed on the products of *MYC* RT-qPCR in AR cells in the control and CTO-treated groups. **F,** Changes in *MYC* mRNA remaining in AR cells after treatment with CTO for 1, 2, 4, 6, and 8 h. G, MeRIP analysis of AR cells with anti-m^6^A antibodies, followed by RT-qPCR analysis of *MYC* mRNA in the control and Devimistat-treated groups. Data represent mean ± SD, n = 5 per group. ****, *P* < 0.0001 in unpaired t-test. **H,** Western blot analysis of HIF-1α in AR cells after treatment with Devimistat (200 μM) for 48 h, and DMKG (5 mM) was added to the culture medium during the last 6 h. Data represent mean ± SD, n = 3 per group. **P* < 0.05; ***P* < 0.01; ****P* < 0.001; *****P* < 0.0001 in one-way ANOVA.

**Figure S6. Prolonged CTO exposure attenuates fatty acid synthesis in PDAC cells.** RT-qPCR analysis of key genes of FAS in AR, AS, PANC-1, MiaPaCa-2, and PUMC-PAAD7, PUMC-PAAD10 cells in the control and CTO (20 μM)-treated groups. Data represent mean ± SD, n = 3 per group. *, *P* < 0.05; **, *P* < 0.01; ***, *P* < 0.001; ****, *P* < 0.0001 in unpaired t-test.

**Figure S7. Long-term CTO treatment reduces nucleic acid precursors and lipids, and c-Myc/HIF-1α were down-regulated without activation of AMPK in PDAC cells. A** and **B,** Heatmaps show LC-MS analysis of nucleic acid precursors (**A**) and lipids (**B**) in AS cells in the control and CTO (20 μM)-treated groups. **C,** Western blot analysis of the AMPK-ACC pathway in AS, AR, PANC-1, MiaPaCa-2, PUMC-PAAD7, and PUMC-PAAD10 cells in the control and CTO-treated groups. **D** and **E,** Western blot analysis of the AMPK-ACC pathway (**D**) and c-Myc/HIF-1α (**E**) in AR cells after treatment with 20 μM CTO for 6, 12, 24, and 48 h. Data represent mean ± SD, n = 3 per group. *, *P* < 0.05; **, *P* < 0.01; ***, *P* < 0.001; ****, *P* < 0.0001 in unpaired t-test. **F,** Cell viability of AR cells after treatment with Compound C for 48 h, with or without CTO (20 μM). Data represent mean ± SD, n = 3 per group. ***, *P* < 0.001 in one-way ANOVA; ns, not significant.

**Figure S8. CTO inhibits PDAC cell growth across multiple models. A** and **B,** CTO suppresses tumor growth in PUMC-PAAD7 (**A**) and PUMC-PAAD10 (**B**) xenograft models in NOD-SCID mice. Data represent mean ± SD, n = 9 per group in PUMC-PAAD7 xenograft models; n = 8 for the control group and n = 7 for the CTO group in PUMC-PAAD10 xenograft models . **, *P* < 0.01; ***, *P* < 0.001; ****, *P* < 0.0001 in two-way ANOVA.

**Figure S9. Sustained CTO exposure reduces c-Myc and 5hmC in PDAC tissues. A,** IHC staining of c-Myc in the control and CTO (90 mg/kg)-treated groups from PUMC-PAAD7 and PUMC-PAAD10 tissues (20×) in NOD-SCID mice; scale bar = 50 µm. **B,** Comparison of the abundance of pyruvate and lactate between the control and CTO (90 mg/kg)-treated groups from AR tissues in BALB/c nude mice. Data represent mean ± SD, n = 3 per group. **C,** IHC staining of 5hmC in the control and CTO (90 mg/kg)-treated groups from Pan-02 tissues (20×) in C57BL/6J mice; scale bar = 50 µm. Data represent mean ± SD, n = 4 per group. *, *P* < 0.05; **, *P* < 0.01 in unpaired t-test; ns, not significant.
