# Supplementary material for: Targeting mitochondrial α-ketoglutarate sequestration disables dual oncogenic drivers and metabolic adaptability in pancreatic ductal adenocarcinoma
Source: Cell Death Dis. 2026 Jun 25;17(1):788. doi: 10.1038/s41419-026-09016-1 (PMC13558746; doi:10.1038/s41419-026-09016-1)
Supplement: Supplementary file 2 [file 41419_2026_9016_MOESM2_ESM.docx]

Table S1 Correlations of YTHDF3 with mitochondrial metabolic enzymes and canonical c-Myc target genes in PAAD cohorts (predominantly PDAC).

| **Gene A** | **Gene B** | **Expression Correlation** | |
| --- | --- | --- | --- |
|  |  | **R** | **P** |
| DLAT | YTHDF3 | 0.72 | 0 |
| DLD |  | 0.65 | 0 |
| DLST |  | 0.58 | 0 |
| OGDH |  | 0.48 | 1.3e−11 |
| G6PD |  | 0.24 | 0.0014 |
| CDK4 |  | 0.36 | 5.9e−07 |
| MCM6 |  | 0.36 | 7.6e−07 |
| SREBF1 |  | 0.13 | 0.089 |
