# Supplementary material for: Targeting mitochondrial α-ketoglutarate sequestration disables dual oncogenic drivers and metabolic adaptability in pancreatic ductal adenocarcinoma
Source: Cell Death Dis. 2026 Jun 25;17(1):788. doi: 10.1038/s41419-026-09016-1 (PMC13558746; doi:10.1038/s41419-026-09016-1)
Supplement: Supplementary file 3 [file 41419_2026_9016_MOESM3_ESM.docx]

Table S2 Correlations between mitochondrial metabolic genes (*OGDH*, *DLST*, *PDHB*, *DLAT,* *DLD*, *SLC25A51*, and *SDHB*) and c-Myc target genes in PAAD cohorts (predominantly PDAC).

| **Gene A** | **Gene B** | **Expression Correlation** | |
| --- | --- | --- | --- |
|  |  | **R** | **P** |
| OGDH | G6PD | 0.35 | 1.9e−06 |
| DLST |  | 0.42 | 5.6e−09 |
| PDHB |  | 0.25 | 0.00069 |
| DLAT |  | 0.5 | 1.2e−12 |
| DLD |  | 0.3 | 4.9e−05 |
| SLC25A51 |  | 0.22 | 0.0037 |
| SDHB |  | 0.55 | 1.3e−15 |
| OGDH | SREBF1 | 0.15 | 0.047 |
| DLST |  | 0.2 | 0.0082 |
| PDHB |  | 0.19 | 0.012 |
| DLAT |  | 0.21 | 0.0043 |
| DLD |  | 0.18 | 0.013 |
| SLC25A51 |  | 0.27 | 0.00033 |
| OGDH | CDK4 | 0.24 | 0.00096 |
| DLST |  | 0.36 | 5.5e−07 |
| PDHB |  | 0.37 | 2.6e−07 |
| DLAT |  | 0.34 | 2.6e−06 |
| DLD |  | 0.29 | 9.7e−05 |
| SLC25A51 |  | 0.36 | 1e−06 |
| SDHB |  | 0.37 | 4.1e−07 |
| OGDH | MCM6 | 0.29 | 8.3e−05 |
| DLST |  | 0.42 | 5.5e−09 |
| PDHB |  | 0.39 | 6.2e−08 |
| DLAT |  | 0.5 | 8.8e−13 |
| DLD |  | 0.31 | 3.2e−05 |
| SLC25A51 |  | 0.27 | 0.00022 |
| SDHB |  | 0.52 | 5.7e−14 |
