# Supplementary material for: Targeting mitochondrial α-ketoglutarate sequestration disables dual oncogenic drivers and metabolic adaptability in pancreatic ductal adenocarcinoma
Source: Cell Death Dis. 2026 Jun 25;17(1):788. doi: 10.1038/s41419-026-09016-1 (PMC13558746; doi:10.1038/s41419-026-09016-1)
Supplement: Supplementary file 4 [file 41419_2026_9016_MOESM4_ESM.docx]

Table S3 Correlations of c-Myc and HIF-1α with glycolytic, pentose phosphate pathway, and fatty acid synthesis genes in PAAD cohorts (predominantly PDAC).

| **Gene A** | **Gene B** | **Expression Correlation** | |
| --- | --- | --- | --- |
|  |  | **R** | **P** |
| LDHA | c-Myc | 0.23 | 0.0018 |
| LDHB |  | 0.42 | 7.2e−09 |
| ENO1 |  | 0.18 | 0.014 |
| G6PD |  | 0.14 | 0.053 |
| PRPS1 |  | 0.15 | 0.051 |
| PRPS2 |  | 0.26 | 0.00055 |
| TKT |  | 0.19 | 0.0091 |
| FASN |  | 0.19 | 0.01 |
| ACACA |  | 0.19 | 0.012 |
| LDHA | HIF-1α | 0.42 | 5.4e−09 |
| SLC2A1 |  | 0.22 | 0.003 |
| LDHB |  | 0.36 | 8e−07 |
| ENO1 |  | 0.34 | 2.9e−06 |
| G6PD |  | 0.49 | 3.5e−12 |
| PRPS1 |  | 0.24 | 0.0015 |
| PRPS2 |  | 0.36 | 9.9e−07 |
| TKT |  | 0.18 | 0.016 |
| ACLY |  | 0.31 | 3.1e−05 |
